# Lattice Parameter Governs Magnetic Ground State Selection in Tsai-Type Intermetallic Compounds

**DOI:** 10.1021/jacs.6c05709

**Published:** 2026-09-30

**Authors:** Farid Labib, Kazuhiro Nawa, Yusuke Nambu, Hiroyuki Takakura, Yoichi Ikeda, Kazuhiko Deguchi, Masato Matsuura, Asuka Ishikawa, Ryoichi Kajimoto, Kazuhiko Ikeuchi, Taku J. Sato, Ryuji Tamura

**Affiliations:** † Research Institute for Science and Technology, 378550 Tokyo University of Science, Tokyo 125-8585, Japan; ‡ Institute of Multidisciplinary Research for Advanced Materials (IMRAM), 13101 Tohoku University, Sendai 980-8577, Japan; § Institute for Integrated Radiation and Nuclear Science, Kyoto University, Osaka 590-0494, Japan; ∥ FOREST, Japan Science and Technology Agency, Kawaguchi, Saitama 332-0012, Japan; ⊥ Division of Applied Physics, Faculty of Engineering, 83969 Hokkaido University, Sapporo 060-8628, Japan; # Institute for Materials Research, Tohoku University, Sendai 980-8577, Japan; ∇ Department of Physics, Graduate School of Science, 12965 Nagoya University, Nagoya 464-8602, Japan; ○ Neutron Science and Technology Center, Comprehensive Research Organization for Science and Society (CROSS), Tokai, Ibaraki 319-1106, Japan; ◆ School of Engineering, Institute of Engineering Innovation, 13143 The University of Tokyo, Tokyo 113-8656, Japan; ¶ Materials and Life Science Division, J-PARC Center, Japan Atomic Energy Agency, Tokai, Ibaraki 319-1195, Japan; ☆ Institute of Materials Structure Science, 226490 High Energy Accelerator Research Organization (KEK), Tsukuba 305-0801, Japan; ◎ Institute for Solid State Physics, The University of Tokyo, Kashiwa 277-8581, Japan; ☼ Department of Materials Science and Technology, Tokyo University of Science, Tokyo 125-8585, Japan

## Abstract

Identifying the key parameters that govern magnetic ground states in complex intermetallics remains a central challenge in materials chemistry. Although the valence-electron concentration (*e*/*a*) has frequently been used to classify magnetism in Tsai-type approximant crystals, its predictive power is limited across different alloy families and constituent elements. Here, we demonstrate that the lattice parameter serves as a unified structural parameter governing magnetic ground state selection in Tsai-type icosahedral compounds. Within the framework presented in this work, magnetic ground states collapse onto a single phase diagram that is independent of alloy family and rare-earth species: antiferromagnetic order emerges above a critical lattice parameter of ∼14.72 Å, whereas ferromagnetic order is stabilized within the intermediate range 14.62 Å ≲ *a* ≲ 14.72 Å. These results indicate that the lattice parameter serves as an experimentally accessible structural parameter that encodes the electronic information traditionally represented by *e*/*a* and thereby provides a unified description of magnetic ground state selection. The trends revealed in the present work provide a useful guideline for identifying compositions that may stabilize previously unexplored magnetic phases, including antiferromagnetic quasicrystals and hedgehog-like magnetic textures.

## Graphical abstract

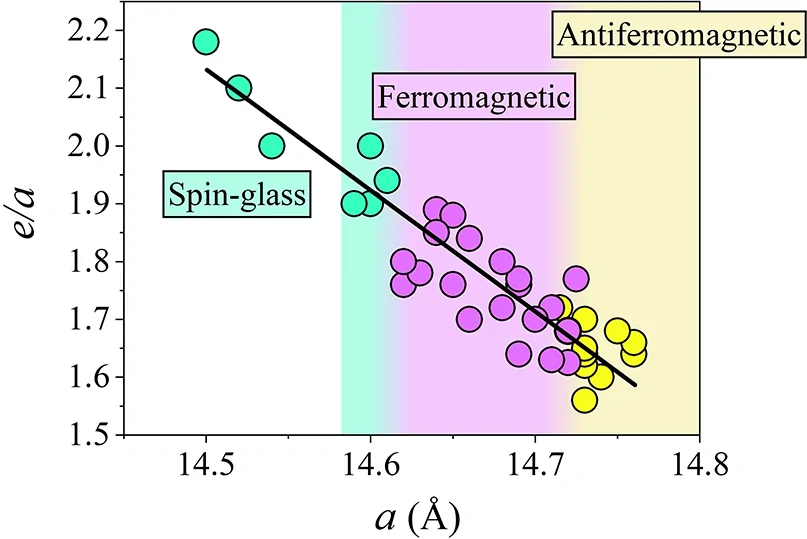

## 1 Introduction

Identifying parameters governing magnetic ground states in complex intermetallic compounds remains a central challenge in materials chemistry. In rare-earth (RE) metallic systems, magnetism arises from the interplay between localized 4*f* moments, conduction-electron–mediated Ruderman–Kittel–Kasuya–Yosida (RKKY) interactions^1–3^ and crystal electric field (CEF) anisotropy.^4,5^ Historically, several chemically tunable parameters have been employed to tune magnetic properties across a wide range of compounds. For example, inspired by the Slater–Pauling principle,^6^ the valence-electron concentration (*e*/*a*) has frequently been used as an empirical guideline for tuning magnetic properties in metallic alloys. The *e*/*a* has also been employed to classify magnetic ground states in typical metallic systems such as Heusler alloys,^7^ quasicrystals and their approximant crystals (ACs).^8–10^

Quasicrystals, first realized in 1982 (published in 1984),^11^ constitute a unique class of solid-state materials exhibiting noncrystallographic rotational symmetries incompatible with three-dimensional translational periodicity. Their structures are built from concentric atomic shells, such as those seen in Tsai-type variants^12^ (see Figure 1). In these compounds, magnetism originates from RE ions occupying the vertices of the icosahedral shell.

**Figure 1:**
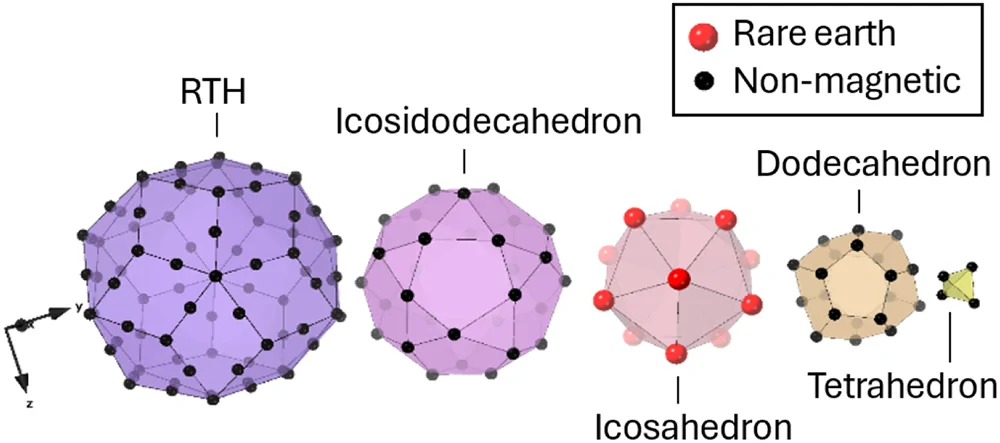
A shell structure of the Tsai-type compounds. From the outermost shell to the center: a rhombic triacontahedron (RTH) with 92 atomic sites, an icosidodecahedron (30 atomic sites), an icosahedron (12 atomic sites), a dodecahedron (20 atomic sites), and an innermost tetrahedron (4 atomic sites).

The emergence of magnetic order in quasicrystals and ACs has recently attracted considerable attention due to their ability to host exotic magnetic states absent in conventional periodic crystals, as suggested by several theoretical studies.^13–18^ This prospect has been reinforced by recent experimental discoveries of long-range ferromagnetic (FM)^19–21^ and antiferromagnetic (AFM)^22^ order in magnetic quasicrystals, as well as a variety of ordered magnetic states in their 1/1 AC counterparts.^23–33^ These findings establish quasicrystal-based intermetallics as a promising platform for exploring emergent magnetism.

Systematic experimental studies of magnetism on Au-based quasicrystals and ACs^8,9^ have identified an empirical relationship between magnetic ground states and the valence-electron concentration (*e*/*a*). Long-range AFM order tends to predominantly appear in the low *e*/*a* region, whereas long-range FM order is typically stabilized at intermediate *e*/*a* values. For larger *e*/*a* values (typically above 1.83–1.9), the magnetic ground state evolves into a spin-glass state.

Here, it is shown that the *e*/*a* lacks universality in predicting magnetic ground states across different alloy families. Instead, we introduce the lattice parameter as a more universal descriptor capturing the structural factors governing magnetic interactions in non-Heisenberg Tsai-type ACs. Through systematic studies of Au–(Al/Ga)–RE (RE = Tb, Dy, Ho) 1/1 ACs, it is revealed that magnetic ground states collapse onto a single lattice parameter phase diagram independent of alloy family and RE species. Within this framework, the whirling AFM order appears only above a critical lattice parameter of approximately 14.72 Å, while the FM order stabilizes within the intermediate range 14.62 Å ≲ *a* ≲ 14.72 Å. These findings establish the lattice parameter as a robust structural parameter governing magnetic ground state selection in Tsai-type intermetallic compounds.

## 2 Experimental Section

Polycrystalline Au–Ga–Dy, Au–Ga–Ho, and Au–Al–Tb 1/1 AC alloys with varying Au, Ga, and Al contents and a constant RE concentration of 14 at. % were synthesized by arc melting under an argon atmosphere. The as-cast ingots were subsequently subjected to isothermal annealing at *T* = 973 K for Au–Ga-based systems and *T* = 1073 K for Au–Al-based systems for 100 h, followed by water quenching.

Among the synthesized samples, selected compositions were investigated for their atomic and magnetic structures using single-crystal X-ray diffraction (SCXRD) and powder neutron diffraction (PND), respectively. Indexing, integration of SCXRD peak intensities, and absorption correction were performed using the CrysAlisPRO software suite (Rigaku Oxford Diffraction, 2018). Structural refinement was carried out using SHELXT^34,35^ for initial model determination and SHELXL^35^ for final least-squares refinement.

Phase identification of all synthesized samples was conducted using powder X-ray diffraction (PXRD) with a Rigaku SmartLab SE diffractometer employing Cu–Kα radiation. Lattice parameters were determined by Le Bail fitting of the PXRD patterns using the JANA2020 software package.^36^

DC magnetic susceptibility measurements were performed under zero-field-cooled (ZFC) and field-cooled (FC) conditions using a superconducting quantum interference device (SQUID) magnetometer (Quantum Design MPMS3). Measurements were conducted in the temperature range 1.8 < *T* < 300 K under applied magnetic fields up to 7 T.

Powder neutron diffraction experiments were carried out using the high-resolution powder diffractometer HERMES installed at the Japan Research Reactor-3 (JRR-3).^37^ Neutrons with a wavelength of λ = 1.34 Å were selected using the Ge(551) reflection.

For the measurements, 3.3 g of Au_68_Ga_18_Dy_14_ and Au_60_Ga_26_Dy_14_ 1/1 AC samples were wrapped in aluminum foil and shaped into thin-walled cylinders with a diameter of 10 mm. The samples were sealed in a vanadium–nickel can filled with He exchange gas. The sample can was mounted on the cold head of a closed-cycle ^4^He refrigerator and cooled to 2 and 15 K during measurements. Absorption correction was numerically performed assuming an ideal thin-walled cylindrical geometry.

Inelastic neutron scattering (INS) experiments were performed using the 4SEASONS spectrometer installed at the Materials and Life Science Experimental Facility (MLF) of the Japan Proton Accelerator Research Complex (J-PARC)^38^ to estimate CEF parameters. The main data set was collected with an incident neutron energy of *E*_*i*_ = 12.7 meV using a Fermi chopper operating at 300 Hz.

For these measurements, 4.1 g of Au_65_Ga_21_Dy_14_ 1/1 AC was wrapped in aluminum foil and formed into a thin-walled cylinder with a diameter of 20 mm. The polycrystalline sample was cooled to 6 K using a top-loading closed-cycle ^4^He refrigerator with He exchange gas. Data reduction and analysis were performed using the Utsusemi software suite,^39^ together with a Fortran-based numerical absorption-correction program.^40^

## 3 Results and Discussion

### 3.1 Data Set Expansion and Structural Scaling in Tsai-Type Approximant Crystals

In the present study, thermally stable Tb-, Dy-, and Ho-based 1/1 ACs with nominal compositions Au_*x*_Al_86–*x*_Tb_14_ (*x* = 68.5, 68.75, and 69), Au_*x*_Ga_86–*x*_Dy_14_ (45 < *x* < 68), and Au_*x*_Ga_86–*x*_Ho_14_ (45 < *x* < 68) were synthesized following a protocol described in section 2. These compositions were deliberately selected to fill gaps in the existing database of magnetic ACs, thereby enabling a more comprehensive exploration of hidden trends governing their magnetic behavior. PXRD patterns in Figure S1 of the Supporting Information exhibit sharp Bragg peaks with their positions being in excellent agreement with those in the calculated pattern using structural details provided elsewhere^29,41^ confirming the high structural quality and purity of the synthesized phases. Among the synthesized 1/1 ACs, representative ones with nominal compositions Au_68_Ga_18_Dy_14_, Au_60_Ga_26_Dy_14_, and Au_62_Ga_24_Ho_14_ are selected for detailed structural analysis using SCXRD measurements, the results of which are provided in Figure S2, Figure S3 and Figure S4 of the Supporting Information, respectively. Crystallographic data, refinement parameters, and details of the final structural models, including atomic coordinates, Wyckoff positions, site occupations, and equivalent isotropic displacement parameters (*U*_eq_) are listed in Tables S1–S6. All refined structural models crystallize in the space group *Im*3̅, forming a body-centered cubic (BCC) lattice in which rhombic triacontahedral (RTH) clusters occupy the cube corners and body center. The RTH cluster comprises a central disordered tetrahedral unit and three concentric inner shells, in agreement with the established prototype Tsai-type structure model described elsewhere.^12^

### 3.2 Correlation between Lattice Parameter and Valence Electron Concentration

Following the successful synthesis of Au_*x*_Al_86–*x*_Tb_14_ (*x* = 68.5, 68.75, and 69), Au_*x*_Ga_86–*x*_Dy_14_ (45 < *x* < 68), and Au_*x*_Ga_86–*x*_Ho_14_ (45 < *x* < 68) 1/1 ACs, their magnetic susceptibilities were systematically investigated, as presented in Figures S5–S7. These measurements substantially expanded the available data set of magnetic Tsai-type compounds^10,23–29,41–43^ as summarized in Table S7 of the Supporting Information. The resulting expanded database enables a comprehensive analysis of the structural and magnetic properties across a broad range of Tsai-type systems.

Figure 2 presents the *e*/*a* ratio as a function of the lattice parameter for Tsai-type compounds listed in Table S7, revealing a nearly monotonic inverse correlation between these quantities. The background colors in Figure 2 indicate the magnetic ground state, with yellow, violet, and green representing AFM, FM, and spin-glass states, respectively. The figure shows that the AFM order emerges only at low *e*/*a* ranges and lattice parameters above approximately 14.72 Å, whereas FM order appears within the intermediate range 14.62 Å ≲ *a* ≲ 14.72 Å. The boundaries between the magnetic ground states in Figure 2 are determined through a comprehensive assessment of macroscopic and microscopic experimental evidence, which will be discussed in the following sections.

**Figure 2:**
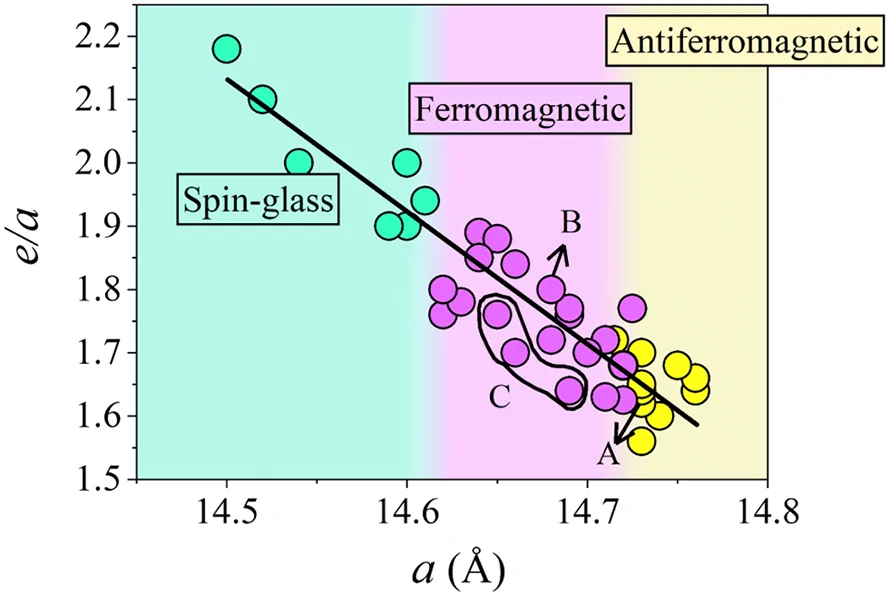
Lattice parameter dependence of *e*/*a* observed for a large number of Tsai-type compounds listed in Table S7 revealing a near-monotonic correspondence between these quantities. The color of the markers indicates their magnetic ground states. The data points indicated by letters A, B and C represent case studies discussed in Section 3.3. The *e*/*a* values were calculated based on Mizutani scale^44^ assigning the following values: Au = 1; Al, Ga, Tb, Dy, and Ho = 3; and Si = 4.

The overall inverse correlation between *e*/*a* and *a* in Figure 2 can be qualitatively understood in terms of valence-driven lattice contraction. Yet the persistence of this monotonic trend across various alloy families and RE species strongly suggests the presence of a common structural origin in Tsai-type systems driven by electronic effects. This trend implies that the lattice parameter may serve not merely as a geometric factor but also as an effective structural descriptor of the underlying electronic state in Tsai-type intermetallics.

In the following section, the existing literature gap on the magnetic properties of Dy- and Ho-based Tsai-type 1/1 ACs is addressed by detailed bulk magnetization and neutron diffraction experiments on selected samples.

### 3.3 Magnetic Orders in Dy- and Ho-Based Tsai-Type 1/1 Approximant Crystals

#### 3.3.1 Dy-Based; Antiferromagnetism

Among the synthesized samples, Au_68_Ga_18_Dy_14_ 1/1 AC exhibiting an AFM order was selected as a representative sample for detailed microscopic analysis. This AFM 1/1 AC has *a* = 14.73 Å and lies within the AFM region in Figure 2 (indicated by an arrow A). The temperature dependence of the dc magnetic susceptibility (*M*/*H*), inverse magnetic susceptibility (*H*/*M*) and the field dependence of magnetization (*M*–*H*) of the sample up to 7 T (Figure 3(a) and (b), respectively), evidence a distinct sharp cusp at *T*_N_ = 6.7 K, a Curie–Weiss temperature *θ*_W_ = +7.3 ± 0.3 K (analyzed within a temperature range of 50 K–300 K), an effective moment *μ*_eff_ = 10.67 *μ*_B_ and a critical metamagnetic transition at approximately *H* = 0.22 T (metamagnetic transition corresponds to a partial reversal of the magnetic moments under strong enough external magnetic field and is a sign of an AFM state controlled by Ising-type anisotropy). Notice that the magnetization curve in Figure 3(b) does not saturate to the full Hund’s-rule moment of Dy^3+^ (10 *μ*_B_/Dy^3+^) even at 7 T. Such unsaturated magnetization has been commonly observed in all non-Heisenberg Tsai-type compounds containing Tb, Dy, and Ho,^19–21,23–26,29^ and reflects the strong CEF-induced magnetic anisotropy that restricts moment rotation under an applied magnetic field (details about the CEF excitation energy will be provided in section 3.4). This interpretation is further supported by magnetic properties of Gd-based Tsai-type compounds, which are close to the Heisenberg limit and exhibit conventional magnetic saturation near the full moment of 7 *μ*_B_/Gd^3+^.^20,30^

**Figure 3:**
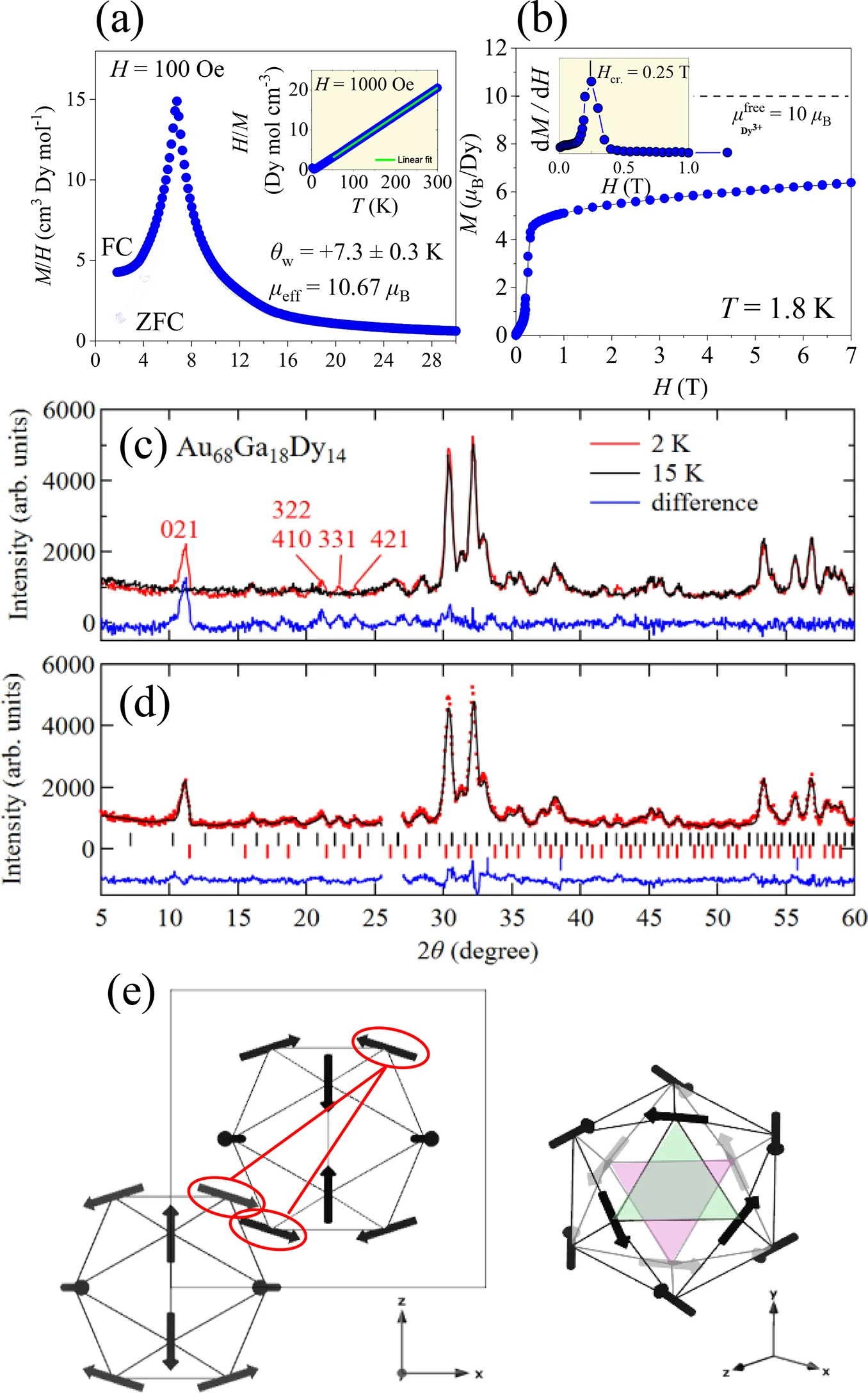
(a) Temperature dependence of the dc magnetic susceptibility (*M*/*H*) and inverse magnetic susceptibility (*H*/*M*) for Au_68_Ga_18_Dy_14_, (b) the field dependence of magnetization (*M*–*H*) of the same compound measured at 1.8 K up to 7 T. (c) PND patterns of the same compound collected at 2 K (red) and 15 K (black). The blue curve represents the difference between the two data sets. (d) Rietveld refinement of Au_68_Ga_18_Dy_14_. The observed, calculated, and difference intensities are shown by red dots, black, and blue curves, respectively. Nuclear and magnetic reflections associated with the antiferromagnetic order are indicated by the upper black and lower red vertical ticks, respectively. Blue vertical ticks denote reflections from the Al foil. The 2*θ* range between 25.5° and 27° was excluded from the refinement to suppress the influence of unknown impurities. (e) Representation of a whirling AFM order in Au_68_Ga_18_Dy_14_ 1/1 AC from different perspectives.

PND patterns of the Au_68_Ga_18_Dy_14_ at *T* = 15 K and *T* = 2 K are compared in Figure 3(c) and (d), respectively. At 2 K, magnetic Bragg reflections appear at indices *hkl* with *h* + *k* + *l* = 2*n* + 1 (*n*: integer), indicating a break of the BCC symmetry of the 1/1 AC by the AFM order. The strongest magnetic Bragg reflection is 021, which appears at 2*θ* = 11.0°. To solve the magnetic structure, Rietveld refinement of the PND pattern is carried out following the approach discussed in refs 23–26 . Here, the structural parameters obtained by SCXRD experiments (listed in TableS4) are used as initial parameters to refine the crystal structures. The refined crystallographic data and structural parameters are summarized in Tables S8–S10 of the Supporting Information.

For constructing the magnetic structure, magnetic representation theory^45^ is applied, which decomposes the magnetic representations into linear combinations of magnetic basis vectors within a single irreducible representation (IR) of the *k*-group, with *k* = (1, 1, 1) associated with the crystallographic space group. The decomposition results and corresponding magnetic basis vectors for all IRs are provided in Tables S11 and S12, listing IRs and the atomic coordinates of the Dy1–Dy12 sites, respectively. The fit at 15 K (Figure S8­(a)) shows good agreement with the experimental data. A fit at 2 K (solid black line in Figure 3(d)) also yields good agreement when the magnetic structure belonging to IR2 is used.

The refined magnetic structure of Au_68_Ga_18_Dy_14_ (Figure 3(e)), represented by the magnetic space group *Ipm*′ – 3′ (204.5, OG setting), is noncoplanar. Due to the presence of 3-fold rotational symmetry, magnetic moments form a whirling configuration around the [111] crystallographic axis, hence denoted a “whirling” AFM order. The magnetic moments are antiparallel at opposite vertices of a single cluster, yielding zero net magnetic moment per cluster. Similarly, moments are antiparallel at equivalent positions in adjacent clusters. In the refined model, the canting angle between the magnetic moments and the pseudo-5-fold axis connecting the center of the cluster to a vertex is 77.7°, and the magnitude of the moment is 5.58(12) *μ*_B_.

#### 3.3.2 Dy-Based; Ferromagnetism

Among the synthesized samples, Au_60_Ga_26_Dy_14_ 1/1 AC is selected as a representative sample exhibiting FM order for detailed microscopic analysis. This 1/1 AC has *a* = 14.68 Å and lies within the FM region in Figure 2 (indicated by arrow B). Temperature dependence of the dc magnetic susceptibility (*M*/*H*) and a field dependent magnetization curve (*M*–*H*) at 1.8 K (Figure 4(a) and (b), respectively) evidence a FM transition at *T*_C_ = 8.6 K. The bifurcation behavior of ZFC and FC susceptibilities in Figure 4(a) arise from the magnetic hysteresis induced from a spontaneous magnetic moment. This is supported by a rapid rise of magnetization with the applied magnetic field below *T*_C_ = 8.6 K, reaching ∼6 *μ*_B_/Dy^3+^ (∼70% of the free-ion moment) at *T* = 1.8 K and *H* = 7 T. The unsaturated magnetization in Figure 4(b) arises from strong CEF-induced magnetic anisotropy, which constrains moment rotation under an applied magnetic field (the microscopic origin and energy scale of this anisotropy are discussed in Section 3.4). The *θ*_W_ value is +6.2 ± 0.2 K (analyzed over the temperature range 50–300 K), and *μ*_eff_ is 10.67 *μ*_B_, close to the theoretical free-ion value for Dy^3+^, $g_{J}\sqrt{J\left(J+1\right)}\mu_{B}$ ,^46^ indicating localization of moments on the Dy^3+^ ions.

PND patterns of Au_60_Ga_26_Dy_14_ at *T* = 2.0 and 15 K (Figure 4(c)), corresponding to below and above *T*_C_ = 8.6 K, respectively, demonstrate the appearance of magnetic reflections with indices *hkl* satisfying *h* + *k* + *l* = *n* (*n*: integer), consistent with the BCC crystallographic symmetry. Rietveld refinement of the PND patterns is carried out following the approach used for the AFM sample earlier. Refined crystallographic data and structural parameters are listed in Tables S8, S13, S14 of the Supporting Information. The fit at 15 K shows good agreement using the structural model (see Figure S8­(b) for the full pattern and Table S13 for refined structural parameters). To solve the magnetic structure, the magnetic modulation vector *k* = (0, 0, 0) is selected since the BCC symmetry is preserved. A fit at 2 K (solid black line in Figure 4(d)) also yields good agreement when the magnetic structure belonging to IR8 is used (see Table S11 for the list of IRs).

Following the details discussed in the Supplementary Table S8, Table S15 and their caption, the best model with the magnetic space group *R*3̅ is selected, as demonstrated in Figure 4(e). In the model, moments on opposite sides of the icosahedron, as well as equivalent moments in neighboring clusters, are aligned in parallel, resulting in a finite net magnetization along a 3-fold direction. The moment magnitude equals 4.61(18) *μ*_B_, and the canting angle relative to the pseudo-5-fold axis is 67.6(2)°.

#### 3.3.3 Dy-Based; Spin-Glass

A standard approach for distinguishing a canonical spin-glass state from a magnetically ordered state is ac magnetic susceptibility measurement.^47^ Owing to the nonequilibrium nature of spin glasses, both the magnitude of the susceptibility and the spin-freezing temperature, *T*_f_, exhibit a characteristic dependence on the measurement frequency (*f*).

Among the synthesized Dy-based 1/1 ACs, Au_55_Ga_31_Dy_14_ and Au_50_Ga_36_Dy_14_ exhibit markedly weaker magnetic susceptibility (Figure S5), reduced field-induced magnetization (Figure S9­(a)), and qualitatively different *θ*_W_ values (Table S7) compared with those of the AFM and FM compounds. These observations indicate a substantial change in the underlying magnetic interactions likely associated with a transition to spin-glass ground state. To investigate this scenario, ac magnetic susceptibility measurements were performed on Au_55_Ga_31_Dy_14_ and compared with that of Au_60_Ga_26_Dy_14_, for which FM order has been established in Section 3.3.2. The two compounds possess lattice parameters of 14.60 and 14.64 Å, respectively.

**Figure 4:**
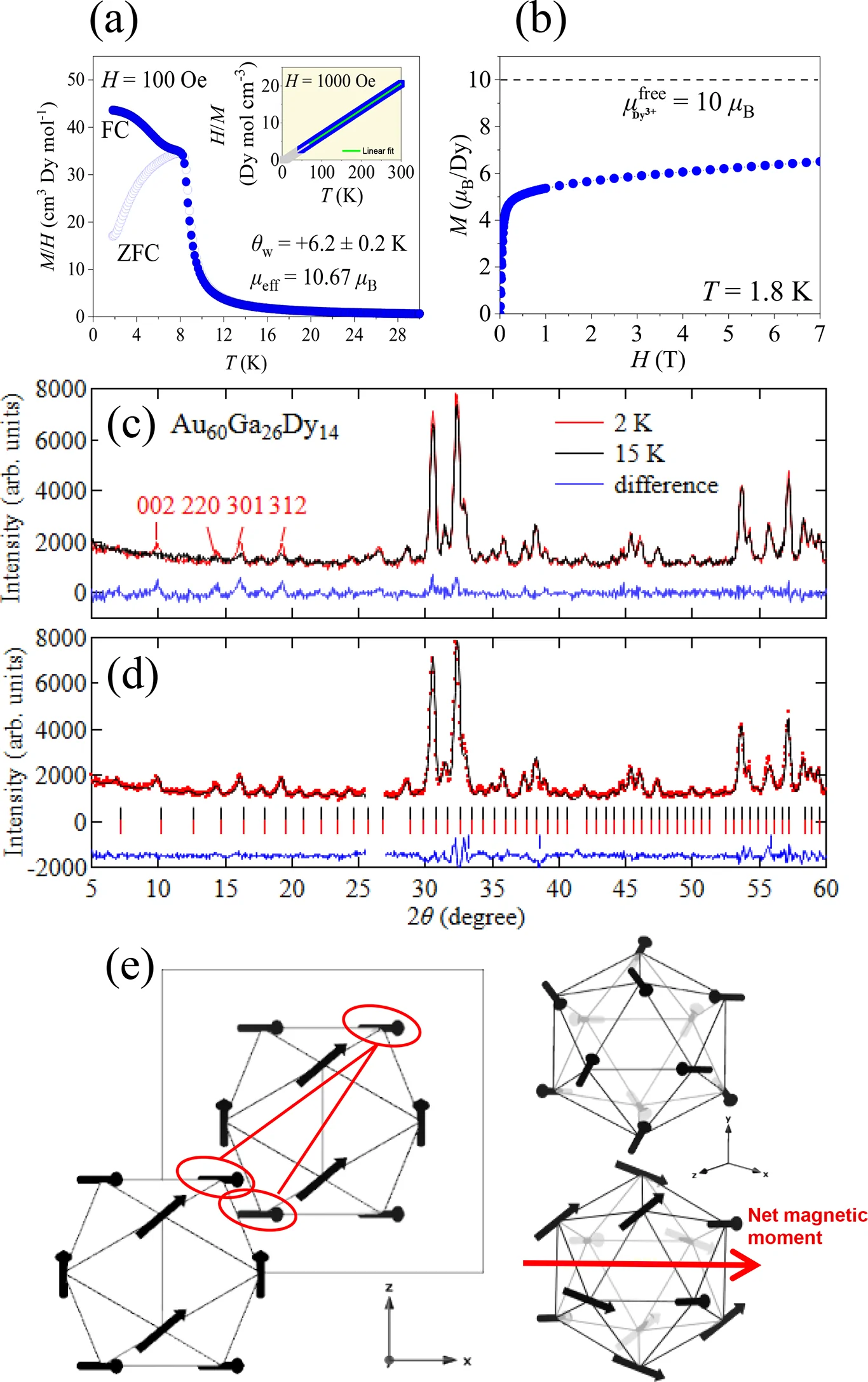
(a) Temperature dependence of the dc magnetic susceptibility (*M*/*H*) and inverse magnetic susceptibility (*H*/*M*) for Au_60_Ga_26_Dy_14_, (b) the field dependence of magnetization (*M*–*H*) of the same compound up to 7 T. (c) PND patterns of the same compound collected at 2 K (red) and 15 K (black). The blue curve represents the difference between the two data sets. (d) The Rietveld refinement of Au_60_Ga_26_Dy_14_, wherein the observed, calculated, and difference intensities are shown by red dots, black, and blue curves, respectively. Nuclear and magnetic reflections associated with the ferromagnetic order are indicated by the upper black and lower red vertical ticks, respectively. Blue vertical ticks denote reflections from the Al foil. The 2*θ* range between 25.5° and 27° was excluded from the refinement to suppress the influence of unknown impurities. (e) Representation of a whirling FM order in Au_60_Ga_26_Dy_14_ 1/1 AC from different perspectives.

As shown in Figure 5(a), Au_55_Ga_31_Dy_14_ exhibits a pronounced frequency dependence of the in-phase ac susceptibility, $\chi_{\mathrm{ac}}^{\prime }$ , whereas Au_60_Ga_26_Dy_14_ (Figure 5(b)) displays an essentially frequency-independent transition characteristic of magnetic order. The transition temperatures in Figure 5 were determined from the minima in the second derivative of $\chi_{\mathrm{ac}}^{\prime }$ . The corresponding Mydosh parameter, *K* = Δ*T*_f_/[*T*_f_Δlog­(*f*)], is estimated to be 0.068 for Au_55_Ga_31_Dy_14_, consistent with canonical spin-glass behavior. This value is comparable to those reported for (LaGd)­Al_2_ (*K* = 0.06), (EuSr)S (*K* = 0.06), and (FeMg)­Cl_2_ (*K* = 0.08),^47^ as well as the Zn_57_Mg_34_Tb_9_ icosahedral quasicrystal (*K* = 0.049)^48^ and the Ga_50_Pd_36_Dy_14_ 2/1 AC (*K* = 0.074).^49^ It should be noted that the weak frequency dispersion of $\chi_{\mathrm{ac}}^{\prime }$ observed below *T*_C_ in FM Au_60_Ga_26_Dy_14_ is likely associated with slow spin relaxation caused by strong single-ion anisotropy, as discussed in Section 3.4. Such behavior is commonly observed in non-Heisenberg Tsai-type systems.^19,20^ A further possible contribution could be the presence of trace amounts of RE atoms at the cluster centers, which has been proposed as a source of weak frequency-dependent behavior in related FM samples.^50^ Based on these ac susceptibility results, *a*  ≈ 14.62 Å (i.e., a value intermediate between those of Au_55_Ga_31_Dy_14_ and Au_60_Ga_26_Dy_14_) is considered as the boundary separating the FM and spin-glass regions.

**Figure 5:**
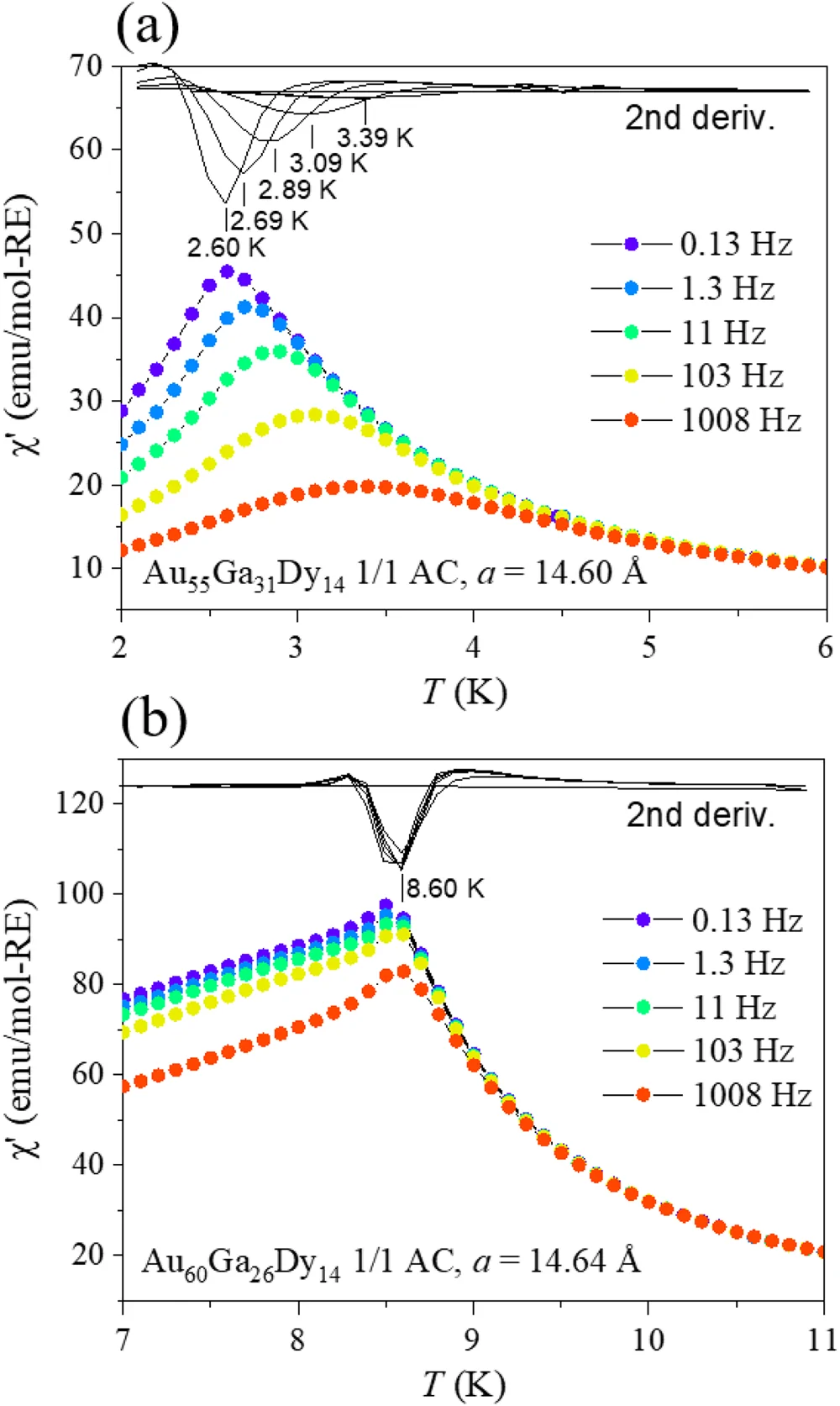
In-phase ac magnetic susceptibility (χ^′^ *_ac_*) of (a) Au_55_Ga_31_Dy_14_ (*a* = 14.60 Å) and (b) Au_60_Ga_26_Dy_14_ (*a* = 14.64 Å) measured over 4 orders of magnitude in frequency (0.13–1008 Hz). The transition temperatures were determined from the minima in the second derivative of χ^′^ *_ac_*.

#### 3.3.4 Ho-Based; Ferromagnetism

In the present study, five new Ho-based 1/1 ACs with nominal compositions Au_*x*_Ga_86–*x*_Ho_14_ (*x* = 50, 55, 62, 65, and 68) are successfully synthesized, among which those with *x* = 62, 65, and 68 are found to exhibit a FM (or ferrimagentic)-like divergence in magnetic susceptibility at *T*_C_ = 3.8–4.9 K (see Figure S6­(a–c)) accompanied by sharp field-induced magnetization curves (Figure S9­(b)). The magnetization of these three 1/1 ACs reach ∼7–8 *μ*_B_/Ho^3+^at 7 T (∼80% of the free-ion moment), consistent with previous reports on Ho-based 1/1 ACs (Au_61.1_Al_25.3_Ho_13.6_ and Au_68.60_Si_16.71_Ho_14.70)_.^28,29^ These 1/1 ACs have *a* = 14.69, 14.66, and 14.65 Å, respectively, and appear within the FM region in Figure 2 (collectively denoted by the letter C).

Neutron diffraction studies on related Ho-based 1/1 ACs reveal noncoplanar magnetic structures with the magnetic space group *R*3̅ and propagation vector *k* = 0 that preserve the BCC symmetry.^29^ The Ho moments on opposite sides of the icosahedral shell (Wyckoff position 24*g*), as well as equivalent moments in neighboring clusters, are aligned in parallel, resulting in a finite net magnetization along a 3-fold direction (Figure 6). Similar to the whirling structures observed in the Tb- and Dy-based counterparts, 3-fold rotational symmetry is preserved. Interestingly, the magnetic moment size depends sensitively on the occupancy of the cluster center. When a cluster center is occupied by a tetrahedron, the ordered moment on the icosahedral shell reaches approximately 9 *μ*_B_,^29^ whereas, when it is partially occupied by Ho atom, its antiparallel coupling with respect to moments locating on the icosahedron shell reduces the ordered moment size to approximately 7.7 *μ*_B_, which gives rise to a ferrimagnetic-like state.^28^

**Figure 6:**
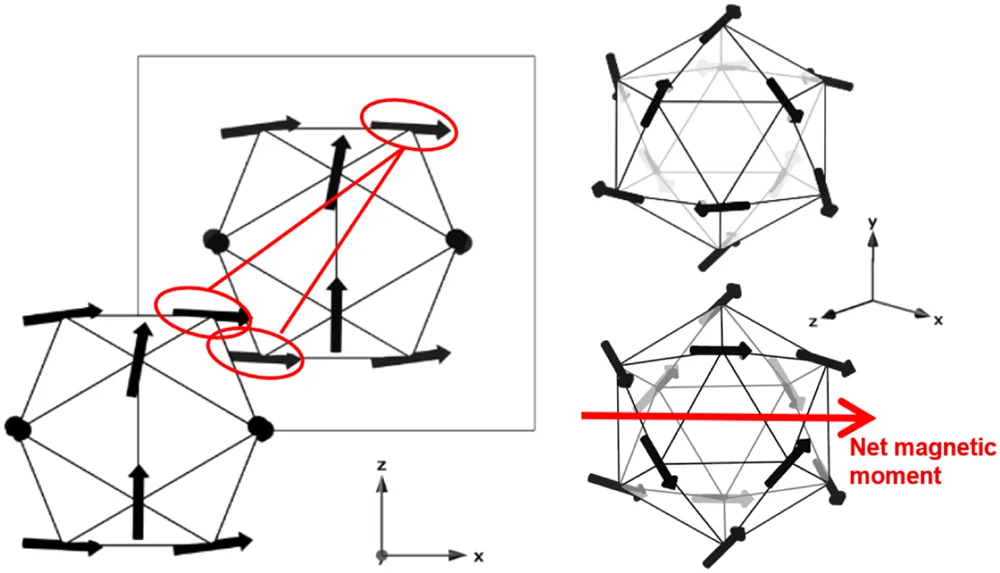
Representation of a whirling FM order in Au_61.1_Al_25.3_Ho_13.6_ 1/1 AC from different perspectives. The magnetic structure visualization was generated using crystallographic and magnetic structure information reported elsewhere.^29^

### 3.4 Crystal Electric Field Effects and Magnetic Anisotropy

The similarity of the magnetic structures observed across non-Heisenberg 1/1 ACs (Figure 3(e), Figure 4(e), and Figure 6) points to eas*y*-axis magnetic anisotropy as a dominant factor governing the RE magnetic moment configurations. Generally, in RE compounds, such magnetic anisotropy originates from CEF splitting of the 4*f J* multiplet. To probe the CEF excitations in Tsai-type 1/1 ACs, the inelastic neutron scattering measurements were performed on a representative FM compound with a nominal composition Au_65_Ga_21_Dy_14_. The inelastic neutron spectra reveal a well-defined CEF excitation consistent with strong uniaxial anisotropy with common characteristics across different RE species, which provides the microscopic basis for classifying the magnetic ground states of these compounds within a unified single phase diagram, as shall be discussed in Section 3.6.

The inelastic neutron spectrum collected at 6 K (Figure 7(a)) reveals a flat excitation at 3.3 meV. Given that the characteristic energy scale of the RKKY interaction extracted from bulk measurements is on the order of ∼0.1 K, as expected from the Weiss temperature,^23–26^ the estimated CEF excitation gap of ∼3 meV (∼30 K) is more than 2 orders of magnitude larger than the exchange interaction. Therefore, the orientations of the magnetic moments in the ordered phase are predominantly fixed by the local CEF-induced uniaxial anisotropy (Ising spins). The wavevector (h) dependence of the integrated intensities at 2–5 meV (Figure 7(b)) follows the trend expected from the magnetic form factor of Dy^3+^, indicating that the flat mode corresponds to crystal field excitations of Dy^3+^. In addition, the energy-transfer dependence of the integrated intensities at 1–2 Å^–1^ measured at several temperatures (Figure 7(c)) shows a peak shift to lower energy transfer with increasing temperature. No significant signal is observed at high energies, except for a weak flat mode at 170 meV (see Figure S10 for details).

**Figure 7:**
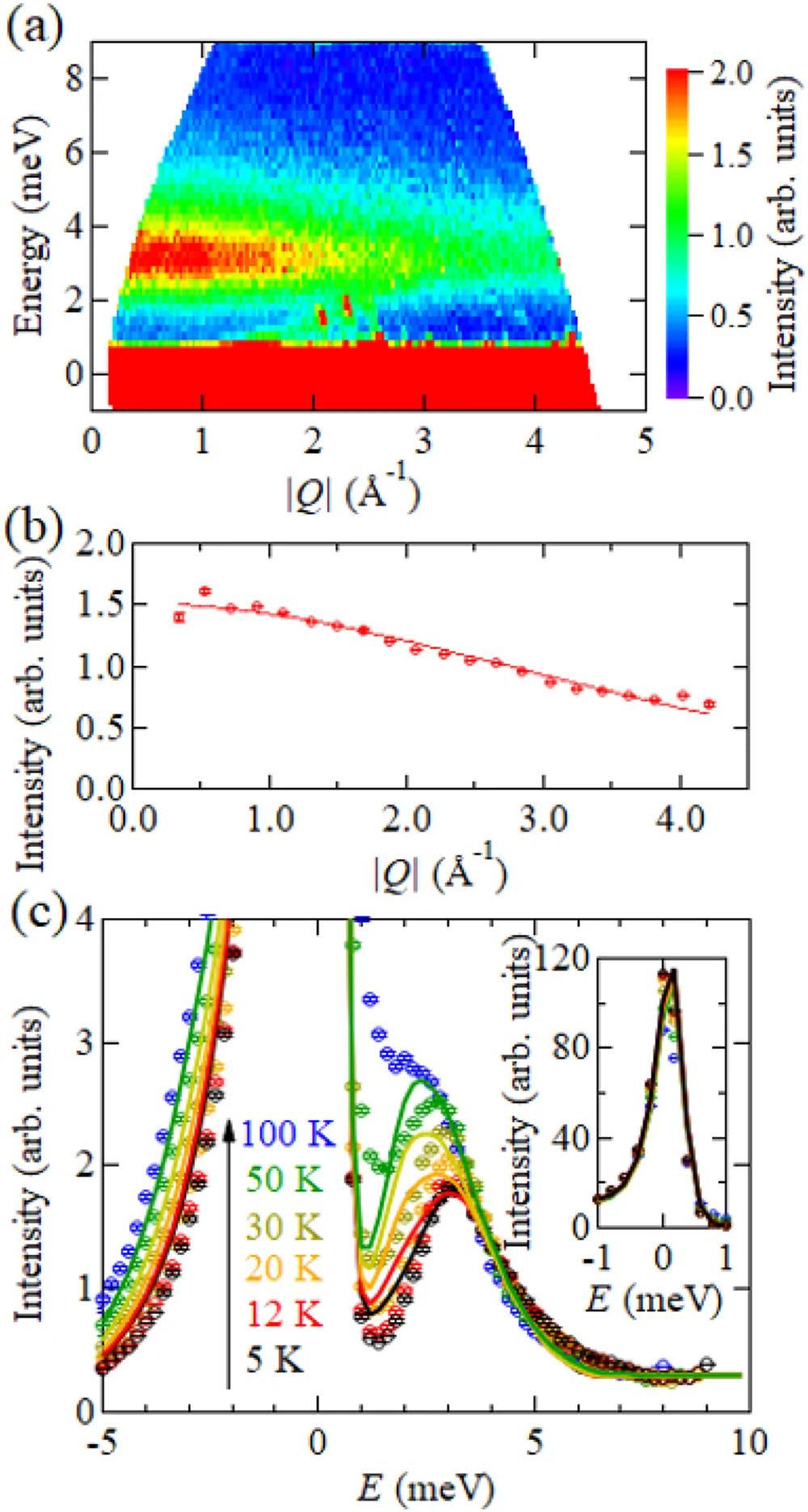
(a) Inelastic neutron scattering spectrum of Au_65_Ga_21_Dy_14_ 1/1 AC collected at 6 K with an incident neutron energy of 12.74 meV. (b) Wavevector dependence of the intensities integrated over the energy range 2–5 meV. The red solid curve represents a fit to the magnetic form factor of Dy^3+^. (c) Energy dependence of the intensities integrated over the wavevector range 1–2 Å^–1^. Solid curves indicate fits based on the point-charge model (see text for details).

To gain quantitative insight into CEF excitations, the inelastic neutron spectrum was analyzed using a point charge model constructed from the refined atomic structure of Au_68_Ga_18_Dy_14_ 1/1 AC. In this model, the electrostatic CEF potential acting on the Dy^3+^ ion is calculated from the surrounding atomic charges, and the corresponding excitation spectrum is obtained by diagonalizing the CEF Hamiltonian within the *J* = 15/2 multiplet of Dy^3+^. Details of the formalism and calculation procedure are provided in the Appendix to the Supporting Information. The model includes three adjustable point-charge parameters (*q*_Au_, *q*_Ga_, and *q*_Dy_), which were optimized to reproduce the experimental inelastic spectrum. The best agreement was obtained for *q*_Au_ = 0.08, *q*_Ga_ = 0.44, and *q*_Dy_ = −0.08, as shown by the solid curves in Figure 7(c). In the paramagnetic temperature range up to 50 K, the calculated spectra reproduce the experimentally observed single excitation peak reasonably well.

Analysis of the resulting CEF parameters indicates that the dominant contributions arise from second-order terms (*k* = 2), leading to a strong uniaxial anisotropy. The ground state is therefore close to the |±15/2⟩ doublet, consistent with an Ising-type character of the Dy^3+^ magnetic moments. Consequently, the inelastic neutron scattering spectrum is dominated by transitions between adjacent CEF doublets, which explains the single peak observed at low temperature and its temperature-dependent evolution.

Using the optimized CEF potential, the principal axis of the magnetic moment distribution was also estimated. The resulting eas*y*-axis direction is broadly consistent with the magnetic moment orientations determined experimentally, indicating that the strong Ising anisotropy of Dy^3+^ originates primarily from the local electrostatic environment. Because the point-charge model is sensitive to the local Au/Ga arrangement around Dy, variations in the local atomic configuration can lead to significant changes in the magnetic anisotropy between the AFM and FM compositions.

### 3.5 Microscopic Origin of Whirling Magnetic Orders in Non-Heisenberg Tsai-Type Compounds

This section provides the microscopic basis for the classification of the magnetic states within a unified scale that shall be discussed in Section 3.6. The similarities in the magnetic structures observed across non-Heisenberg Tsai-type compounds containing RE = Tb^3+^,^23–27^ Dy^3+^ (Figure 3(e) and Figure 4(e)) and Ho^3+^ (Figure 6) indicate that whirling AFM and FM orders persist in non-Heisenberg systems. The whirling AFM and FM orders are represented by the magnetic space groups *Ipm*′ – 3′ (204.5, OG setting) and *R*3̅, respectively. Table 1 summarizes the microscopic details of the whirling FM and AFM orders across non-Heisenberg Tsai-type 1/1 ACs, including those revealed in the present work, listing chemical compositions, lattice parameters, magnetic space groups, the magnitude of magnetic moment, and canting angles of the magnetic moments relative to the pseudo-5-fold axis (*θ*).

**Table 1:** Magnitudes and Canting Angles of the Magnetic Moments with Respect to the Pseudo-Fivefold Axis Are Summarized for the Ground State Magnetic Structure of Various 1/1 ACs Studied by Neutron Diffraction to Date^a^

				Moment size (μ_B_)			
	Compound	*a* (Å)	Magnetic space group	RE1	RE2	RE3	canting angle θ (°)
Tb^3+^	Au_70_Al_16_Tb_14_ (AFM)^24^	14.74	*Ipm*′ – 3′	7.77(2)	7.77(2)	–	86.0, 86.0
	Au_72_Al_14_Tb_14_ (AFM)^26^	14.73	*Ipm*′ – 3′	7.50(3)	7.50(3)	–	86.0, 86.0
	Au_65_Ga_21_Tb_14_ (AFM)^23^	14.73	*Ipm*′ – 3′	7.86(1)	7.86(1)	–	85.0, 85.0
	Au_70_Si_17_Tb_13_ (FM)^27^	14.73^b^	*R*3̅	6.90(0)	6.90(0)	–	77.5, 82.0
	Au_64_Ga_22_Tb_14_ (AFM/FM)^25^	14.71	*Ipm*′ – 3′/*R*3̅	5.58(2)	5.58(2)	–	50.0, 50.0
	Au_60_Ga_26_Tb_14_ (FM)^25^	14.69	*R*3̅	5.58(2)	5.58(2)	–	85.6, 85.6
	Au_65.58_Si_20.78_Tb_13.64_ (FM^c^)^28^	14.64^b^	*R*3̅	8.53(2)	9.02(2)	–	69.5, 73.3
	Au_69.87_Si_16.26_Tb_13.87_ ^d^ (FM^c^)^28^	14.62^b^	*R*3̅	3.57(0)	5.59(2)	8.66(6)	46.4, 68.1
Dy^3+^	Au_68_Ga_18_Dy_14_ (AFM) — this work	14.73	*Ipm*′ – 3′	5.58(1)	5.58(1)	–	77.7, 77.7
	Au_60_Ga_26_Dy_14_ (FM) — this work	14.68	*R*3̅	4.61(2)	4.61(2)	–	67.6, 67.1
Ho^3+^	Au_61.1_Al_25.3_Ho_13.6_ (FM^c^)^29^	14.63^b^	*R*3̅	9.22(8)	9.14(8)	–	54.8, 61.1
	Au_68.60_Si_16.71_Ho_14.70_ ^d^ (FM^c^)^28^	14.62^b^	*R*3̅	7.71(7)	7.74(2)	0.85(4)	56.8, 61.5

a The lattice parameter *a* of each compound is also listed. The estimated uncertainty in the calculated canting angles is ±0.1°. The magnetic ground state is indicated in parentheses aftereach composition; “AFM” and “FM” denote antiferromagnetic and ferromagnetic ground states, respectively. For some 1/1 ACs^28,29^ two or more inequivalent magnetic sublattices (RE1 and RE2 sites forming icosahedra and RE3 sites at the cluster center) have been reported, resulting in distinct canting angles. In the other reports^23–27^ RE1 and RE2 are assumed to be equivalent with identical moment magnitudes, yielding similar canting angles.

b These lattice parameters were determined at low temperature; the corresponding room-temperature values could be slightly larger.

c In the *R*3̅ structure, the two sublattices, RE1 and RE2, arise from the magnetic ordering. Because the net magnetic moments of the two sublattices are nearly parallel to each other, we classify this state as a ferromagnetic (FM) structure. This magnetic structure is equivalent to the “ferrimagnetic structure” described in some previous reports.^27,29^ The magnetic moments at RE3 sites are nearly antiparallel to RE1 and RE2 sites if they exist.

d The atomic structure contains an RE atom at the cluster center (RE3), which holds a magnetic moment antiparallel to the rest of the REs on the icosahedron (RE1–RE2).

Table 1 is informative from various aspects. First, it clearly indicates that the orientation of the anisotropy axis can vary slightly among different RE ions. In Tb-based FM and AFM compounds, the magnetic moments align almost perpendicular to the pseudo-5-fold directions of the icosahedral RE clusters, forming a canting angle of *θ* ∼ 86°.^23–26^ This angle does not contradict expectations from point-charge analyses using structural models of Tb-based 1/1 ACs elsewhere,^51^ which predict that the magnetic easy axis lies nearly perpendicular to the pseudo-5-fold direction and can be continuously rotated toward the pseudo-5-fold axis depending on the local CEF environment. Notably, *θ* systematically decreases for heavier RE elements: Dy-based samples exhibit *θ* = 67.6–77.7°, and Ho-based counterparts show *θ* = 54.7–61.4°.^28,29^ This trend in magnetic moment orientation may originate from differences in the reduced matrix elements of the RE ions as well as subtle variations in the local electrostatic environment surrounding the RE ions (see eq A3 and A4 in the Appendix to the Supporting Information).

Moreover, Table 1 indicates that certain 1/1 ACs exhibit smaller magnetic moment sizes than others. One category includes the so-called pseudo-Tsai-type systems, in which the structure is naturally contracted due to the partial replacement of tetrahedra by RE atoms at the cluster center.^28^ Such structural contraction modifies the exchange interactions between localized 4*f* moments mediated by the RKKY mechanism, contributing to reduced moment sizes.

Furthermore, as appears in Table 1, the moment size of the Dy-based 1/1 ACs is reduced compared to other non-Heisenberg RE variants. We speculate that this behavior may originate from the Kramers degeneracy inherent to Dy^3+^ ions (4*f* ^9^, *J* = 15/2), although a microscopic mechanism and the possible correlation remain open questions. The reduced magnetic moment size is also expected to lower the magnitude of the metamagnetic transition field *H*_cri_ in the *M*–*H* curves. Note that, in general, the magnitude of *H*_cri_ is affected by a variety of parameters including the magnetic moment size, the magnitude of magnetic interactions (represented by the Curie–Weiss temperature), or even the canting angles of the magnetic moments from pseudo-5-fold direction, all of which are smaller in Dy-based 1/1 ACs compared to their Tb-based counterparts (Table 1). Lower *H*_cri_ values in the AFM Dy-based 1/1 ACs are indeed confirmed experimentally in Figure S11, which compares *M*–*H* curves of Dy- and Tb-based AFM 1/1 ACs.

To gain further insight into the magnetic interactions underlying the whirling magnetic orders, the angular distributions of magnetic-moment pairs (*φ*, in degrees) in the AFM and FM structures of Au–Ga–Tb and Au–Ga–Dy 1/1 ACs are compared in Figure 8. The analysis considers 324 RE–RE pairs between 5.0 and 10.0 Å, encompassing the first-, second-, and third-nearest-neighbor interactions (*J*_1_, *J*_2_, and *J*_3_, respectively). A schematic representation of these interaction pathways around a representative magnetic moment is shown in Figure 8(a). Within this distance range, both *J*_1_ and *J*_2_ comprise intra- and intercluster interactions, whereas *J*_3_ is predominantly intracluster. Interestingly, both whirling FM and AFM orders exhibit predominantly FM-like alignments (*φ* < 90°) for second-nearest-neighbor pairs (Figure 8(b)). In contrast, their nearest-neighbor correlations differ significantly: nearest-neighbor pairs are predominantly AFM-like (*φ* > 90°) in the AFM structure, whereas they are mainly FM-like (*φ* < 90°) in the FM structure. These results indicate that both whirling AFM and FM states are governed by FM second-nearest-neighbor interactions, while the sign of the nearest-neighbor interaction determines whether the magnetic ground state adopts an AFM or FM configuration.

**Figure 8:**
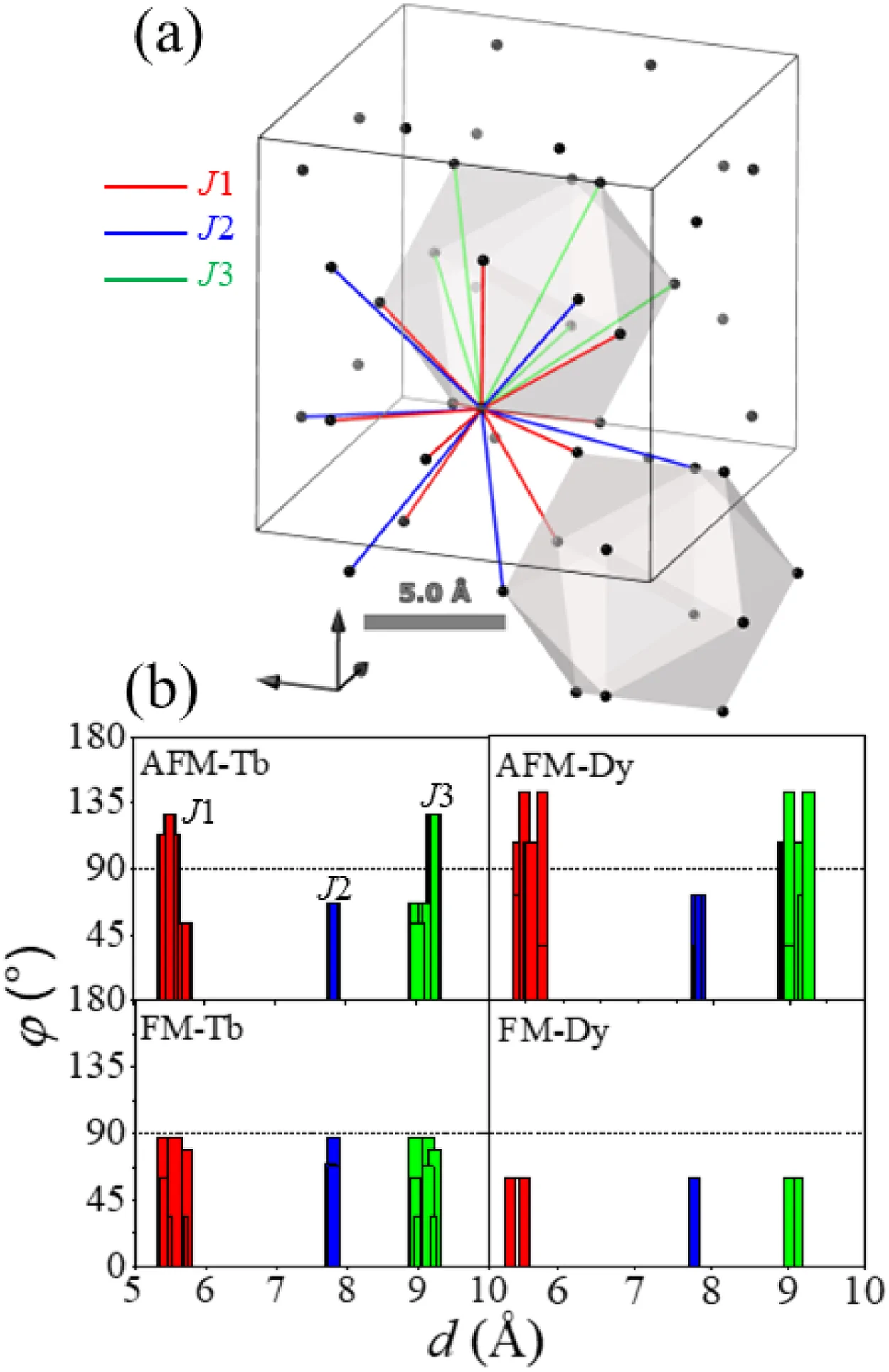
(a) Schematic illustration of the first-, second-, and third-nearest-neighbor interactions (*J*_1_, *J*_2_, and *J*_3_, respectively) surrounding a representative RE magnetic moment in the prototype 1/1 Tsai-type AC structure. (b) Angular distributions (in degrees) of magnetic-moment pairs (*φ*) in the Tb- and Dy-based whirling AFM and FM phases as a function of the RE–RE separation.

In addition, the similarity of the inelastic neutron scattering spectrum of Dy^3+^ ions in Figure 7(a) to that of Tb^3+^ in ref 27 suggests a comparable distribution of electric charge around the RE atoms in Tsai-type compounds. If the electric charge distribution around the RE atoms were similar, the *q*_*kq*_ terms in eq A1 in the Appendix to the Supporting Information would also be similar. The reduced matrix element of the RE ions therefore accounts for the difference in the CEF potential. For both Tb^3+^ and Dy^3+^ ions, the dominant *q*_20_ < 0 term leads to a second-order uniaxial CEF contribution of the form $H_{0}=-DJ_{z}^{2}\left(D>0\right)$ , originating from the negative second-order reduced matrix element ⟨*J*||*c* ^(2)^||*J*⟩ < 0. Ho^3+^ ions also possess a negative second-order reduced matrix element. Hence, it is reasonable to deduce that non-Heisenberg 1/1 ACs, including Tb, Dy, and Ho, share a similar uniaxial anisotropy. This interpretation is supported by a recent theoretical point-charge analysis of Dy-based Tsai-type 1/1 ACs,^52^ which revealed that local moment configurations arising from the cluster environment closely resemble those reported for their Tb-based counterparts.^14,53^ These results unequivocally suggest that whirling magnetic orders are commonly stabilized by the same microscopic origin: “*intracluster FM interactions*” and “u*niaxial magnetic anisotropy of the magnetic moments*”.

### 3.6 Lattice-Parameter as a Descriptor of Magnetic Ground States

Figure 9(a) summarizes the lattice-parameter dependence of the magnetic transition temperatures (*T*_C_, *T*_N_, and *T*_f_) and the corresponding magnetic ground states (distinguished by different background colors) across a wide range of Au–SM–RE (SM = Al, Ga, Si; RE = Tb, Dy, Ho) Tsai-type compounds. The figure reveals a clear universal trend: irrespective of chemical composition or RE species, the whirling AFM state emerges only when the lattice parameter exceeds a critical threshold of approximately 14.72 Å, whereas the FM state is stabilized within the intermediate range 14.62 Å ≲ *a* ≲ 14.72 Å. Below this range, long-range magnetic order is suppressed and a spin-glass state emerges.

**Figure 9:**
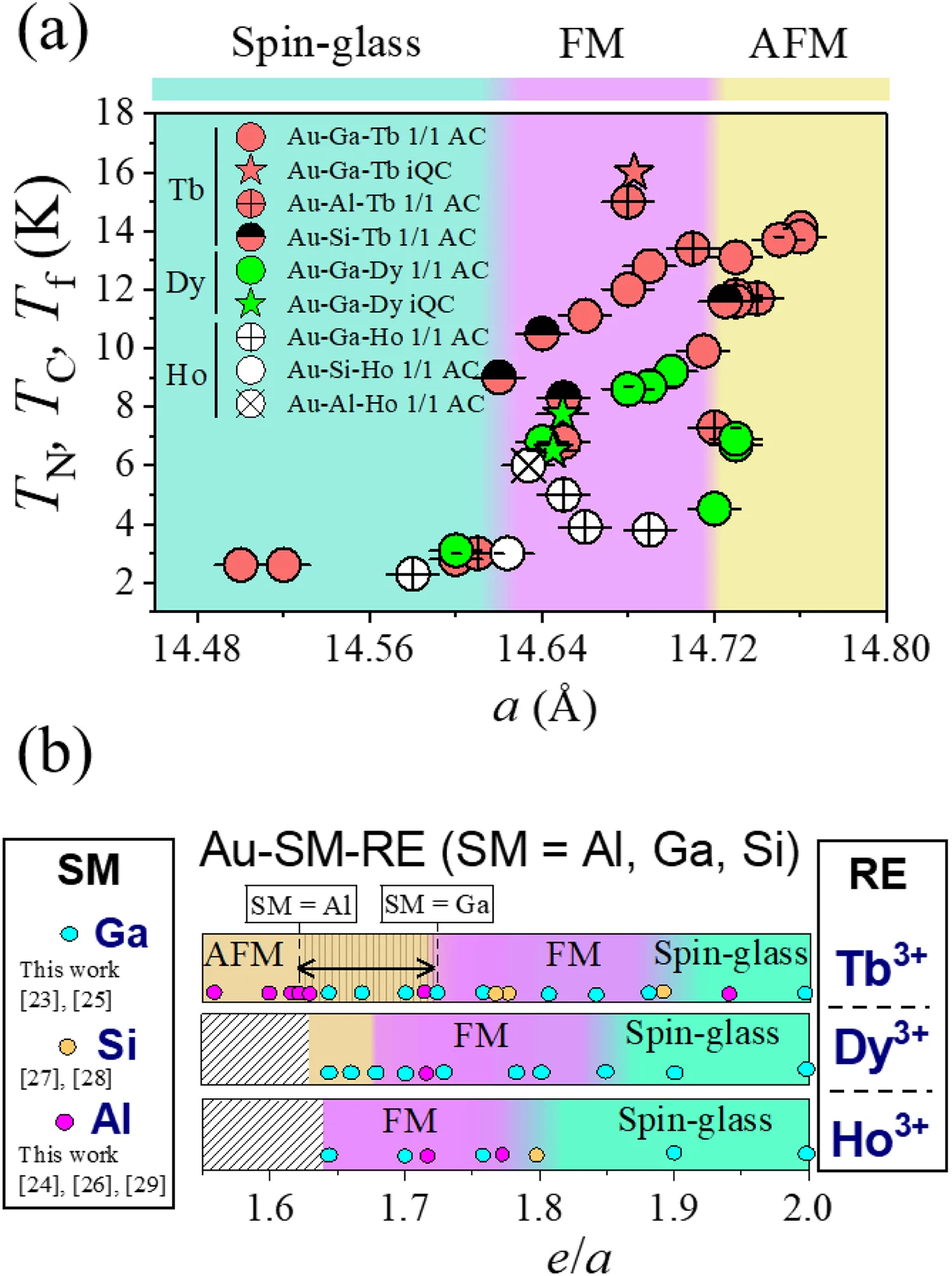
(a) Magnetic phase diagram of Tsai-type non-Heisenberg Au–SM–RE (SM = Al, Ga, Si; RE = Tb, Dy, and Ho) compounds, showing the dependence of *T*_C_, *T*_N_, and *T*_f_ (Curie, Néel, and freezing temperatures, respectively) and the corresponding magnetic ground states. The green, pink, and yellow background colors denote the spin-glass, FM, and AFM regimes, respectively. The *T*_N_ values of Au_66_Ga_20_Dy_14_, Au_68.75_Al_17.25_Tb_14_, and Au_64_Ga_22_Tb_14_ 1/1 ACs exhibiting successive FM/AFM transitions are shown. (b) Magnetic phase diagram of Tsai-type non-Heisenberg Au–SM–RE (SM = Al, Ga, Si; RE = Tb, Dy, and Ho) 1/1 ACs in the *e*/*a* space ranging within 1.64–2.00. Shaded regions in the *e*/*a* space indicate the experimental unavailability of single-phase 1/1 ACs due to chemical instability. Dashed lines in the case of RE = Tb denote the FM/AFM phase boundaries for SM = Al and Ga. The *e*/*a* values were calculated based on Mizutani scale^44^ assigning the following values: Au = 1; Al, Ga, Tb, Dy, and Ho = 3; and Si = 4.

In Figure 9(a), the FM–AFM phase boundary is located at *a* ≈ 14.72 Å through the identification of the Au_66_Ga_20_Dy_14_, Au_68.75_Al_17.25_Tb_14_, and Au_64_Ga_22_Tb_14_ 1/1 ACs. These compounds possess nearly identical lattice parameters in a range of ∼14.71–14.72 Å and exhibit successive FM and AFM transitions in their magnetic susceptibility (see Figure S5­(c), Figure S7­(b), and ref 25). In the last compound, the presence of both FM and AFM orders has been confirmed via neutron diffraction experiment.^25^ Importantly, all three compounds display pronounced dip in their ordering temperatures (*T*_C_ or *T*_N_) relative to neighboring compositions within the same alloy families (only *T*_N_ values of these compounds are shown in Figure 9(a)). Such a reduction of the ordering temperature is indicative of enhanced competition between FM and AFM exchange interactions near the phase boundary. These observations highlight the dominant role of the lattice parameter in governing the magnetic ground states of non-Heisenberg Tsai-type systems.

The power of the lattice-parameter-based framework in governing magnetic ground states becomes particularly evident when compared with the conventional *e*/*a* classification. Figure 9(b) plots the magnetic ground states of Tsai-type compounds as a function of *e*/*a* using an extensive data set compiled from refs 23–26,28,29,54 together with the results of the present study. The figure clearly demonstrates a systematic shift of ground states toward lower *e*/*a* values upon replacing Tb^3+^ (*J* = 6) with Dy^3+^ (*J* = 15/2) and subsequently Ho^3+^ (*J* = 8), or upon replacing the SM element from Al to Ga or Si. The marker colors in Figure 9(b) denote the SM species (green for Ga, violet for Al, and yellow for Si). Notably, in the *e*/*a* parameter space, an AFM phase region appears only in the Tb- and Dy-based systems, while it is entirely absent in the Ho-based counterparts. Furthermore, in the Tb-based 1/1 ACs with SM = Ga, an AFM region spans a relatively broad range of *e*/*a* values (1.64 < *e*/*a* < 1.73), whereas it narrows significantly to 1.64 < *e*/*a* < 1.67 in Dy-based systems. Importantly, when the same data set is plotted against the lattice parameter, the data collapse onto a single unified phase diagram with well-defined phase boundaries (Figure 9(a)). These observations indicate that the lattice parameter serves as a unified and experimentally accessible structural descriptor for magnetic ground state selection in Tsai-type compounds, whereas the *e*/*a* acts only as an indirect control parameter through its correlation with the lattice parameter.

It is worth noting that the lattice parameters of FM Au–Ga–RE (RE = Tb, Dy) quasicrystals^20,21^ fall within the FM region in Figure 9(a) (indicated by star symbols). Although the number of magnetic quasicrystals currently available remains too limited to extend the universality of the present phase diagram beyond 1/1 ACs to icosahedral quasicrystals, this consistency suggests that the same underlying mechanism may govern magnetic ground-state selection in all Tsai-type icosahedral compounds. Here, the lattice parameters in quasicrystals are estimated from the six-dimensional lattice parameters *a*_6D_. This conversion is based on the well-established higher-dimensional description^55,56^ which treats quasicrystals and ACs as different projections of a common six-dimensional structure. Accordingly, the *a* and the *a*_6D_ are related by^57^

1 $$a=\sqrt{\frac{2}{2+\tau }}\left(1+\tau \right)a_{6D}$$

where *τ* is the golden mean (*τ* ≈ 1.618).

The governing role of the lattice parameter on magnetism indicates that electronic structures of ACs and quasicrystals do not solely depend on *e*/*a*. In Tsai-type compounds, the exchange interaction between moments is mediated by conduction electrons through the RKKY mechanism. In its asymptotic form, the RKKY interaction is given by

2 $$J_{\mathrm{RKKY}}\left(r\right)\propto \frac{\cos\left(2k_{F}r\right)}{r^{3}}$$

where *r* is the distance between magnetic moments and *k*_F_ is the Fermi wavevector. The nearly monotonic scaling of *a* with *e*/*a* in Figure 2 suggests that the lattice parameter captures the underlying electronic conditions that govern the entire network of RKKY interactions. The appearance of well-defined magnetic phase boundaries when the data are projected onto the lattice parameter, and their deviation when projected onto the *e*/*a* axis in Figure 2, demonstrates that the lattice parameter provides a more comprehensive descriptor of the electronic conditions relevant to RKKY interactions. This is particularly evident for isovalent elemental substitutions (e.g., Tb → Dy or Ga → Al), where the average number of valence electrons and *e*/*a* remain nearly unchanged. The elemental substitution reconstructs the whole electronic structure, leading to the shift of *k*_F_ despite the same *e*/*a*. As a result, the magnetic phase boundaries in the *e*/*a*-based phase diagram (Figure 9(b)) shift depending on the constituent elements, whereas they remain remarkably robust in the lattice parameter space (Figure 9(a)).

Lastly, the strong influence of the lattice parameter on physical properties is not unique to the present RE-based systems. In Yb-based Tsai-type ACs and quasicrystals, the lattice parameter systematically controls the mean Yb valence, with a critical value of *a*_c_ ≈ 14.42 Å separating the magnetic Yb^3+^ and nonmagnetic Yb^2+^ states and marking the onset of quantum-critical valence fluctuations.^58^ This observation further highlights the close relationship between crystal structure and emergent electronic and magnetic phenomena in Tsai-type compounds.

In conclusion, we have identified the lattice parameter as a unified structural parameter governing magnetic ground state selection in non-Heisenberg Tsai-type compounds. Unlike the conventional*e/a* framework, magnetic ground states collapse onto a single lattice-parameter phase diagram characterized by well-defined FM, AFM, and spin-glass regions. Although a complete microscopic understanding of this relationship remains to be established, one aspect of the observed universality can already be identified, that is, the lattice parameter is not an independently tunable degree of freedom but is closely linked to the electronic structure, as evidenced by the nearly monotonic scaling of *a* with *e*/*a* in Figure 2. In other words, the lattice parameter captures the underlying electronic conditions governing the entire network of RKKY interactions.

The findings of the present work further suggest a systematic change of magnetic moment direction with lattice parameter. Extrapolating this trend suggests that lattice contraction may be a practical pathway toward realization of theoretically predicted hedgehog-like magnetic texture,^14^ in which *θ* approaches zero. Moreover, it has been revealed that AFM order in non-Heisenberg Tsai-type 1/1 ACs emerges only for lattice parameters exceeding *a* ≈ 14.72 Å. This correlation may serve as a useful guideline for identifying compositions capable of stabilizing AFM order in non-Heisenberg quasicrystals, which has not yet been experimentally observed. More broadly, the present work demonstrates that the lattice parameter serves as a unified and experimentally accessible structural descriptor for magnetic ground state selection in Tsai-type compounds.

## Supplementary Material



## Notes

Powder X-ray diffraction patterns of the synthesized samples; refined crystal structures and crystallographic data obtained by single-crystal X-ray diffraction for selected 1/1 approximant crystals; temperature- and field-dependent magnetic susceptibility and magnetization data; powder neutron diffraction patterns, structural and magnetic refinement parameters, and representation analysis; an inelastic neutron scattering spectrum; compositional, structural, electronic, and magnetic parameters used to construct Figure 2; Appendix, which provides details of the point-charge crystal electric field calculations for Dy^3+^, including the treatment of Au/Ga chemical disorder, the calculation of the inelastic neutron scattering intensity and CEF-level distribution, the optimized effective-charge parameters, and the determination of the magnetic eas*y*-axis direction (PDF)
