# Supplementary material for: Lattice Parameter Governs Magnetic Ground State Selection in Tsai-Type Intermetallic Compounds
Source: J Am Chem Soc. 2026 Sep 30;148(41):44019–32. doi: 10.1021/jacs.6c05709 (PMC13693384; doi:10.1021/jacs.6c05709)
Supplement: Supplementary file 1 [file ja6c05709_si_001.pdf]

Supporting Information

Lattice Parameter Governs Magnetic Ground State Selection in Tsai-Type Intermetallic Compounds

Farid Labib[1*], Kazuhiro Nawa[2*], Yusuke Nambu[3,4], Hiroyuki Takakura[5], Yoichi Ikeda[6], Kazuhiko Deguchi[7], Masato Matsuura[8], Asuka Ishikawa[9], Ryoichi Kajimoto[10], Kazuhiko Ikeuchi[11], Taku J. Sato[2,12] and Ryuji Tamura[13]

1 Research Institute of Science and Technology, Tokyo University of Science, Tokyo 125-8585, Japan
2 Institute of Multidisciplinary Research for Advanced Materials (IMRAM), Tohoku University, Sendai 980-8577, Japan
3 Institute for Integrated Radiation and Nuclear Science, Kyoto University, Osaka 590-0494, Japan
4 FOREST, Japan Science and Technology Agency, Kawaguchi, Saitama 332-0012, Japan
5 Division of Applied Physics, Faculty of Engineering, Hokkaido University, Sapporo 060-8628, Japan
6 Institute for Materials Research, Tohoku University, Sendai 980-8577, Japan
7 Department of Physics, Graduate School of Science, Nagoya University, Nagoya 464-8602, Japan
8 Neutron Science and Technology Center, Comprehensive Research Organization for Science and Society (CROSS), Tokai, Ibaraki 319-1106, Japan
9 School of Engineering, Institute of Engineering Innovation, The University of Tokyo, Tokyo 113-8656, Japan
10 Materials and Life Science Division, J-PARC Center, Japan Atomic Energy Agency, Tokai, Ibaraki 319-1195, Japan
11 Institute of Materials Structure Science, High Energy Accelerator Research Organization (KEK), Tsukuba 305-0801, Japan
12 Institute for Solid State Physics, The University of Tokyo, Kashiwa 277-8581, Japan
13 Department of Materials Science and Technology, Tokyo University of Science, Tokyo 125-8585, Japan

Email:
labib.farid@rs.tus.ac.jp
knawa@tohoku.ac.jp

Figures Legend

Figure S1. Powder X-ray diffraction patterns of (a) $Au_xAl_{86-x}Dy_{14}$ ($45 < x < 68$), (b) $Au_xAl_{86-x}Ho_{14}$ ($45 < x < 68$), and (c) $Au_xAl_{86-x}Tb_{14}$ (x = 68.5, 68.75, and 69) 1/1 approximant crystals (ACs). The bottom pattern in each panel corresponds to the calculated diffraction profile obtained from the refined structural models of $Au_{68}Ga_{18}Dy_{14}$ (Fig. S2; Tables S1 and S4), $Au_{61.1}Al_{25.3}Ho_{13.6}$[1], and $Au_{49}Al_{36}Tm_{15}$[2].

Figure S2. Refined crystal structure of the $Au_{68}Ga_{18}Dy_{14}$ 1/1 AC.

Figure S3. Refined crystal structure of the $Au_{60}Ga_{26}Dy_{14}$ 1/1 AC.

Figure S4. Refined crystal structure of the $Au_{62}Ga_{24}Ho_{14}$ 1/1 AC.

Figure S5. Low-temperature magnetic susceptibility (M/H) of (a) $Au_{68}Ga_{18}Dy_{14}$, (b) $Au_{67.5}Ga_{18.5}Dy_{14}$, (c) $Au_{66}Ga_{20}Dy_{14}$, (d) $Au_{64}Ga_{22}Dy_{14}$, (e) $Au_{61.5}Ga_{24.5}Dy_{14}$, (f) $Au_{60}Ga_{26}Dy_{14}$, (g) $Au_{57.5}Ga_{28.5}Dy_{14}$, (h) $Au_{55}Ga_{31}Dy_{14}$, and (i) $Au_{50}Ga_{36}Dy_{14}$ measured under field-cooled (FC) and zero-field-cooled (ZFC) conditions at H = 100 Oe. Insets show the corresponding inverse magnetic susceptibility (H/M) measured under ZFC conditions at H = 1000 Oe.

Figure S6. Low-temperature M/H of (a) $Au_{68}Ga_{18}Ho_{14}$, (b) $Au_{65}Ga_{21}Ho_{14}$, (c) $Au_{62}Ga_{24}Ho_{14}$, (d) $Au_{55}Ga_{31}Ho_{14}$, and (e) $Au_{50}Ga_{36}Ho_{14}$ measured under FC and ZFC conditions at H = 100 Oe. Insets show the corresponding H/M measured under ZFC conditions at H = 1000 Oe.

Figure S7. Low-temperature M/H of (a) $Au_{69}Al_{17}Tb_{14}$, (b) $Au_{68.75}Al_{17.25}Tb_{14}$, and (c) $Au_{68.5}Al_{17.5}Tb_{14}$ measured under FC and ZFC conditions at H = 100 Oe. Insets show the corresponding H/M measured under ZFC conditions at H = 1000 Oe.

Figure S8. Powder neutron diffraction patterns of (a) $Au_{68}Ga_{18}Dy_{14}$ and (b) $Au_{60}Ga_{26}Dy_{14}$ 1/1 ACs collected at 15 K over a wide 2θ range.

Figure S9. Magnetization curves of (a) $Au_{68-x}Ga_{18+x}Dy_{14}$ (x = 0, 3, 13, and 18) and $Au_{68-x}Ga_{18+x}Ho_{14}$ (x = 0, 3, 6, 13, 18) 1/1 ACs measured at 1.8 K up to μ_0H = 7 T.

Figure S10. Inelastic neutron scattering spectrum of $Au_{65}Ga_{21}Dy_{14}$ collected at 5 K using an incident neutron energy of 307 meV.

Figure S11. Comparison of the field dependence of magnetization in antiferromagnetic Au–Ga–Tb and Au–Ga–Dy 1/1 ACs.

Figure S12. Distribution of Dy crystal electric field (CEF) levels (n = 1, 2, …, 16) obtained from 5000 randomly generated atomic configurations. The lowest two levels correspond to the ground-state Kramers doublet.

Table Legends

Table S1. Crystallographic data and refinement parameters obtained from single-crystal X-ray diffraction (SCXRD) analysis of $Au_{68}Ga_{18}Dy_{14}$ 1/1 AC.

Table S2. Crystallographic data and refinement parameters obtained from SCXRD analysis of $Au_{60}Ga_{26}Dy_{14}$ 1/1 AC.

Table S3. Crystallographic data and refinement parameters obtained from SCXRD analysis of $Au_{62}Ga_{24}Ho_{14}$ 1/1 AC.

Table S4. Atomic coordinates, Wyckoff positions, site occupancies, and equivalent isotropic displacement parameters ($U_{eq.}$) of $Au_{68}Ga_{18}Dy_{14}$ refined from SCXRD data.

Table S5. Atomic coordinates, Wyckoff positions, site occupancies, and equivalent isotropic displacement parameters ($U_{eq.}$) of $Au_{60}Ga_{26}Dy_{14}$ refined from SCXRD data.

Table S6. Atomic coordinates, Wyckoff positions, site occupancies, and equivalent isotropic displacement parameters ($U_{eq.}$) of $Au_{62}Ga_{24}Ho_{14}$ refined from SCXRD data.

Table S7. Chemical composition, average electron concentration (*e/a*), lattice parameter, magnetic ground state, magnetic transition temperature (T_N, T_C and T_f), Curie–Weiss temperature (θ_w), and references used to construct Fig. 2 of the main text.

Table S8. Refined crystallographic parameters obtained from powder neutron diffraction (PND) measurements of $Au_{68}Ga_{18}Dy_{14}$ and $Au_{60}Ga_{26}Dy_{14}$ collected at 2 and 15 K.

Table S9. Structural parameters of $Au_{68}Ga_{18}Dy_{14}$ determined from PND data collected at 15 K.

Table S10. Structural parameters of $Au_{68}Ga_{18}Dy_{14}$ determined from PND data collected at 2 K.

Table S11. Irreducible representations for the Dy sites in $Au_{68}Ga_{18}Dy_{14}$ and $Au_{60}Ga_{26}Dy_{14}$ 1/1 ACs.

Table S12. Atomic coordinates of the Dy1–Dy12 sites in $Au_{68}Ga_{18}Dy_{14}$ and $Au_{60}Ga_{26}Dy_{14}$ 1/1 ACs.

Table S13. Structural parameters of $Au_{60}Ga_{26}Dy_{14}$ determined from PND data collected at 15 K.

Table S14. Structural parameters of $Au_{60}Ga_{26}Dy_{14}$ determined from PND data collected at 2 K.

Table S15. Refined crystallographic parameters for two candidate magnetic structure models.

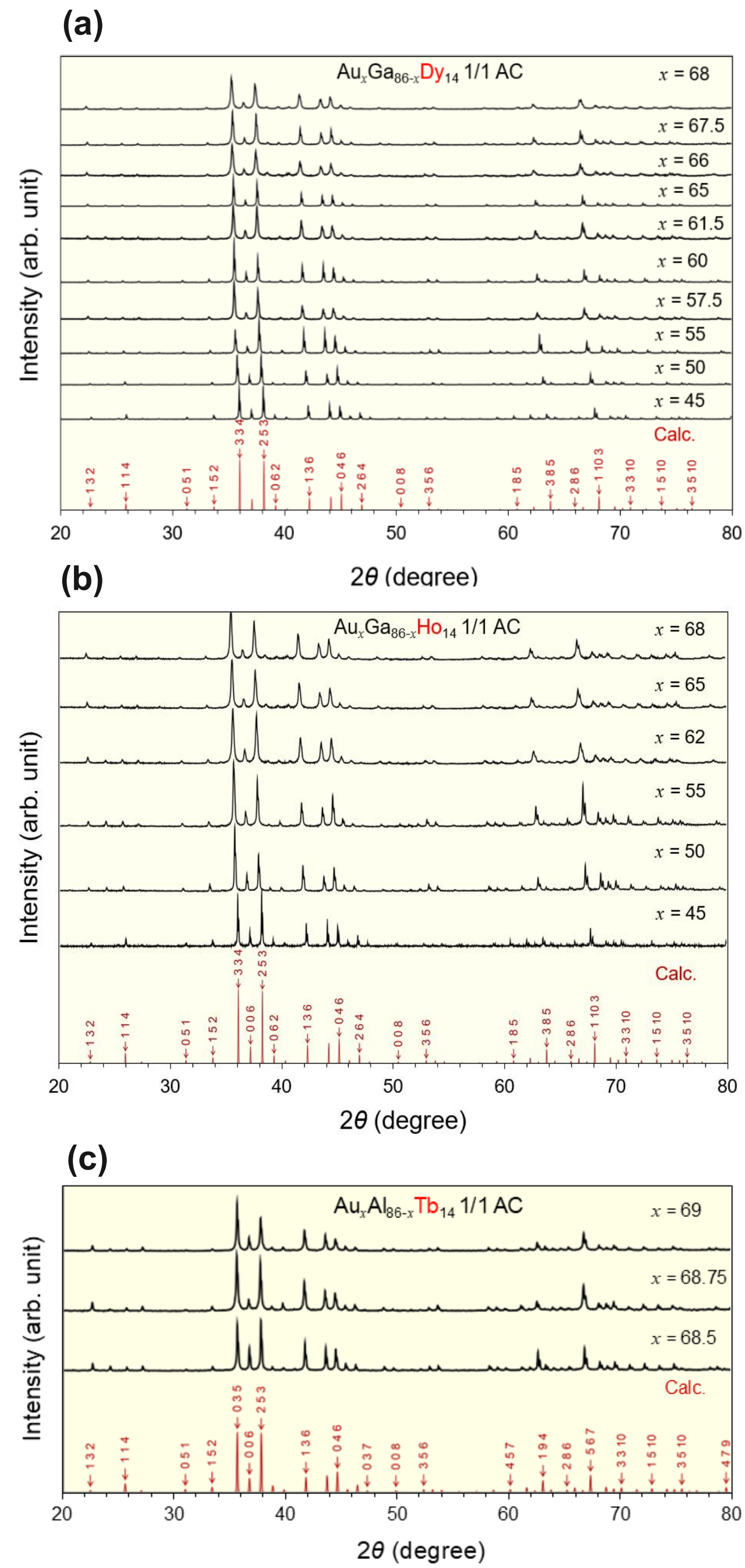

Fig. S1. Powder x-ray diffraction patterns of (a) $Au_xGa_{86-x}Dy_{14}$ ($45 < x < 68$), (b) $Au_xGa_{86-x}Ho_{14}$ ($45 < x < 68$) and $Au_xAl_{86-x}Tb_{14}$ (x = 68.5, 68.75 and 69) 1/1 ACs. The patterns at the bottom correspond to the calculated one obtained from the refined atomic structure models of the $Au_{68}Ga_{18}Dy_{14}$ 1/1 AC (Fig. S2; Tables S1 and S4), $Au_{61.1}Al_{25.3}Ho_{13.6}$ [1] and $Au_{49}Al_{36}Tm_{15}$[2].

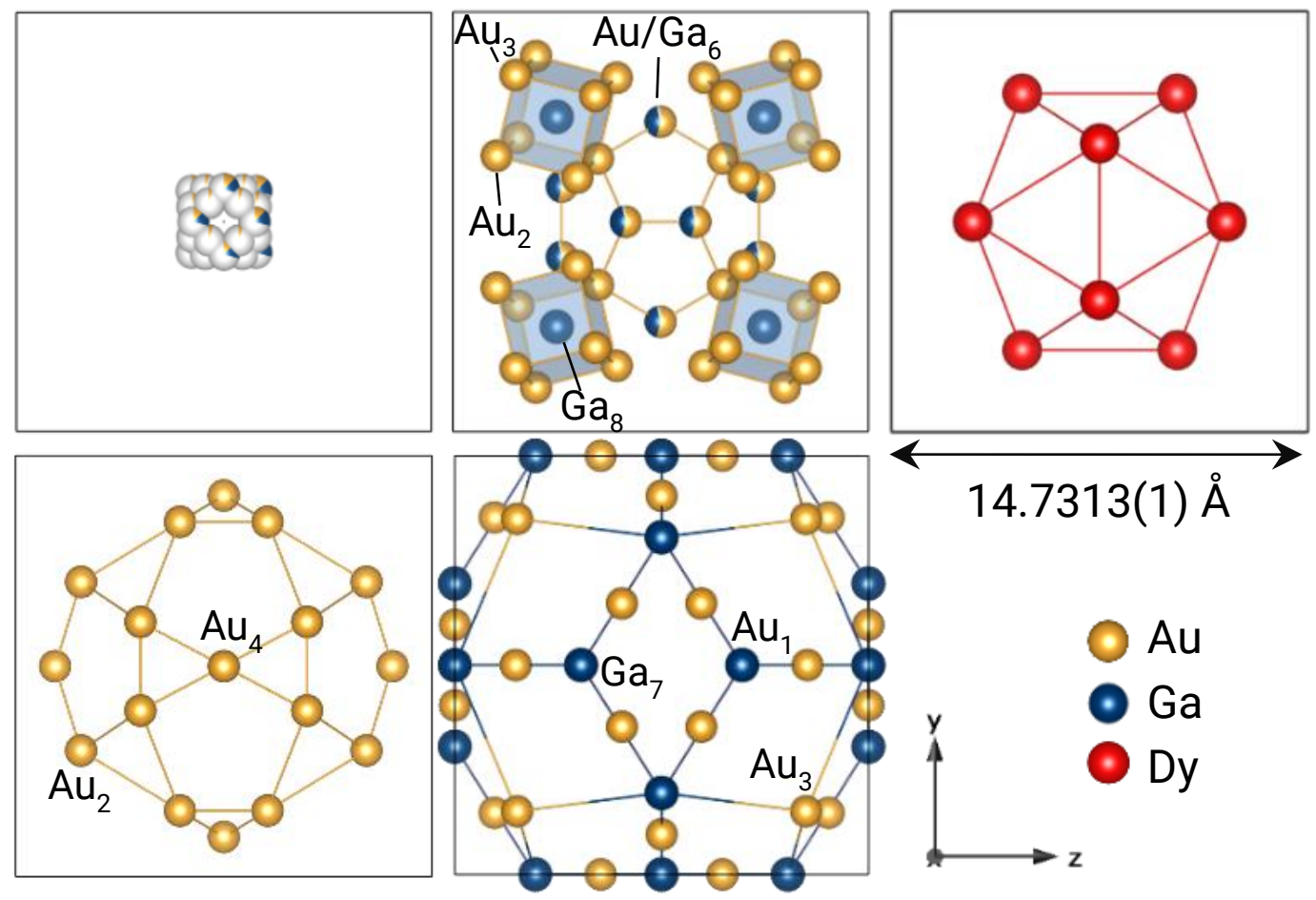

Fig. S2. The successive sequence of atomic shells of the $Au_{68}Ga_{18}Dy_{14}$ 1/1 AC. The innermost unit (top left corner) comprised of orientationally disordered tetrahedron. The vertices of dodecahedron (top middle) are occupied by Au and Ga while the centers of eight cube-shaped interstices connecting adjacent dodecahedra along 3-fold axes are occupied by Ga interstices (Ga8 site). The icosahedron (top right) and icosidodecahedron (bottom left) are exclusively occupied by Dy and Au, respectively. The vertices and mid-edge positions of the rhombic triacontahedron (RTH) in the bottom middle panel are occupied by Ga and Au atoms, respectively.

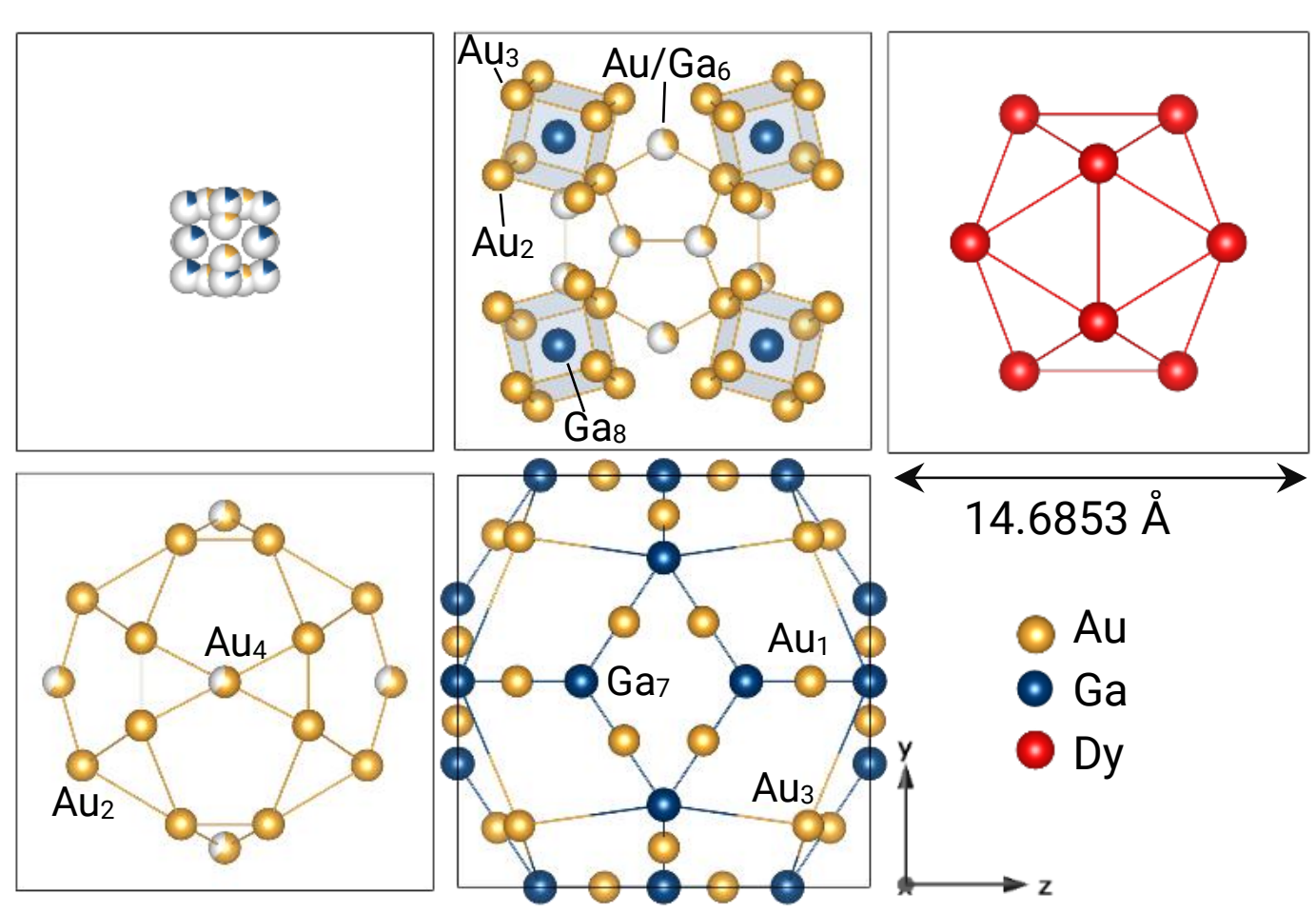

Fig. S3. The successive sequence of atomic shells of the $Au_{60}Ga_{26}Dy_{14}$ 1/1 AC; the innermost unit (top left corner) comprised of orientationally disordered tetrahedron. The vertices of dodecahedron (top middle) are occupied by Au while the centers of eight cube-shaped interstices connecting adjacent dodecahedra along 3-fold axes are occupied by Ga interstices (Ga8 site). The icosahedron (top right) and icosidodecahedron (bottom left) are exclusively occupied by Dy and Au, respectively. The vertices and mid-edge positions of the rhombic triacontahedron (RTH) in the bottom middle panel are fully occupied by Ga and Au atoms, respectively.

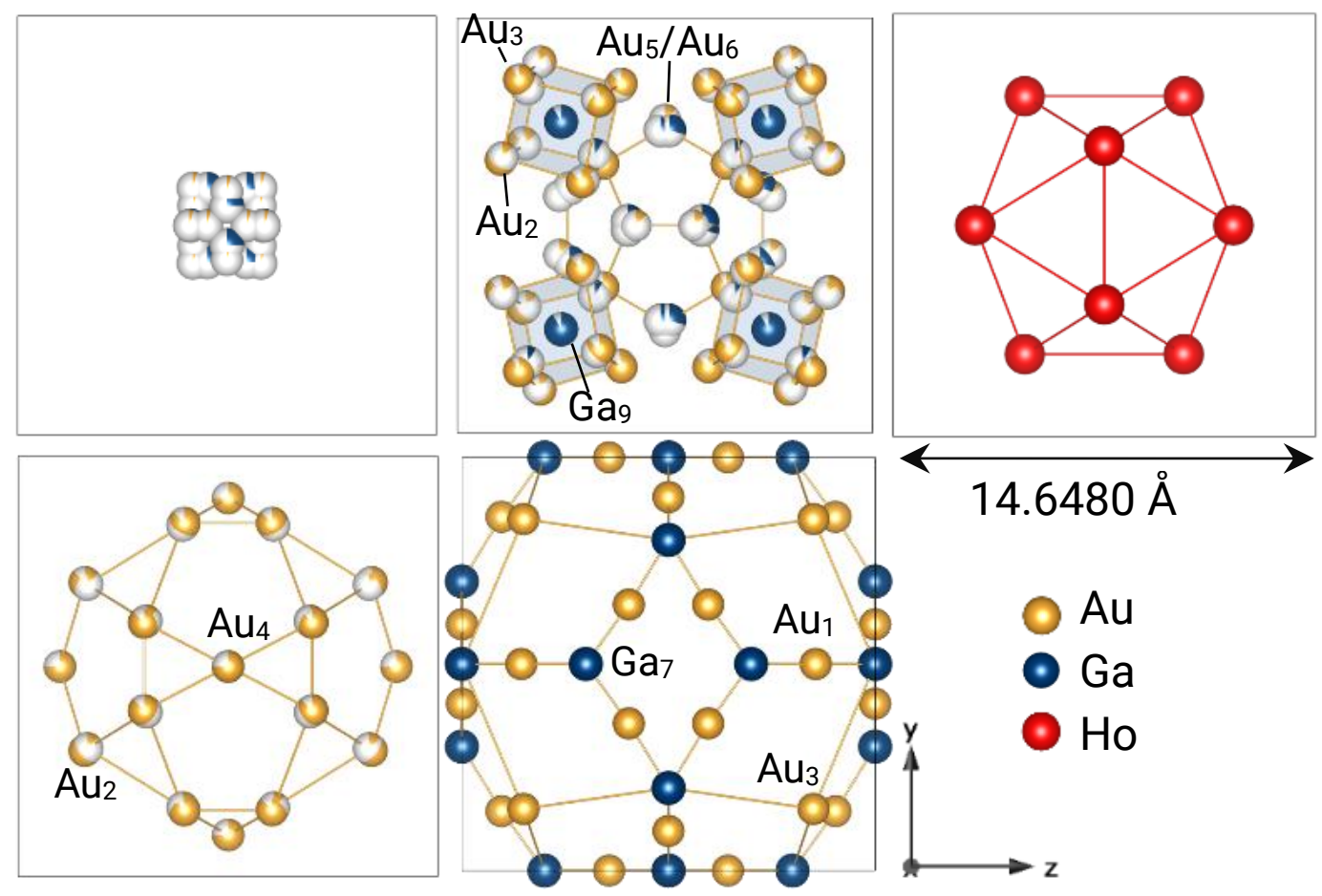

Fig. S4. The successive sequence of atomic shells of the $Au_{62}Ga_{24}Ho_{14}$ 1/1 AC; the innermost unit (top left corner) comprised of orientationally disordered tetrahedron. The vertices of dodecahedron (top middle) are occupied by Au while the centers of eight cube-shaped interstices connecting adjacent dodecahedra along 3-fold axes are occupied by Ga interstices (Ga9 site). The icosahedron (top right) and icosidodecahedron (bottom left) are exclusively occupied by Ho and Au, respectively. The vertices and mid-edge positions of the rhombic triacontahedron (RTH) in the bottom middle panel are fully occupied by Ga and Au atoms, respectively.

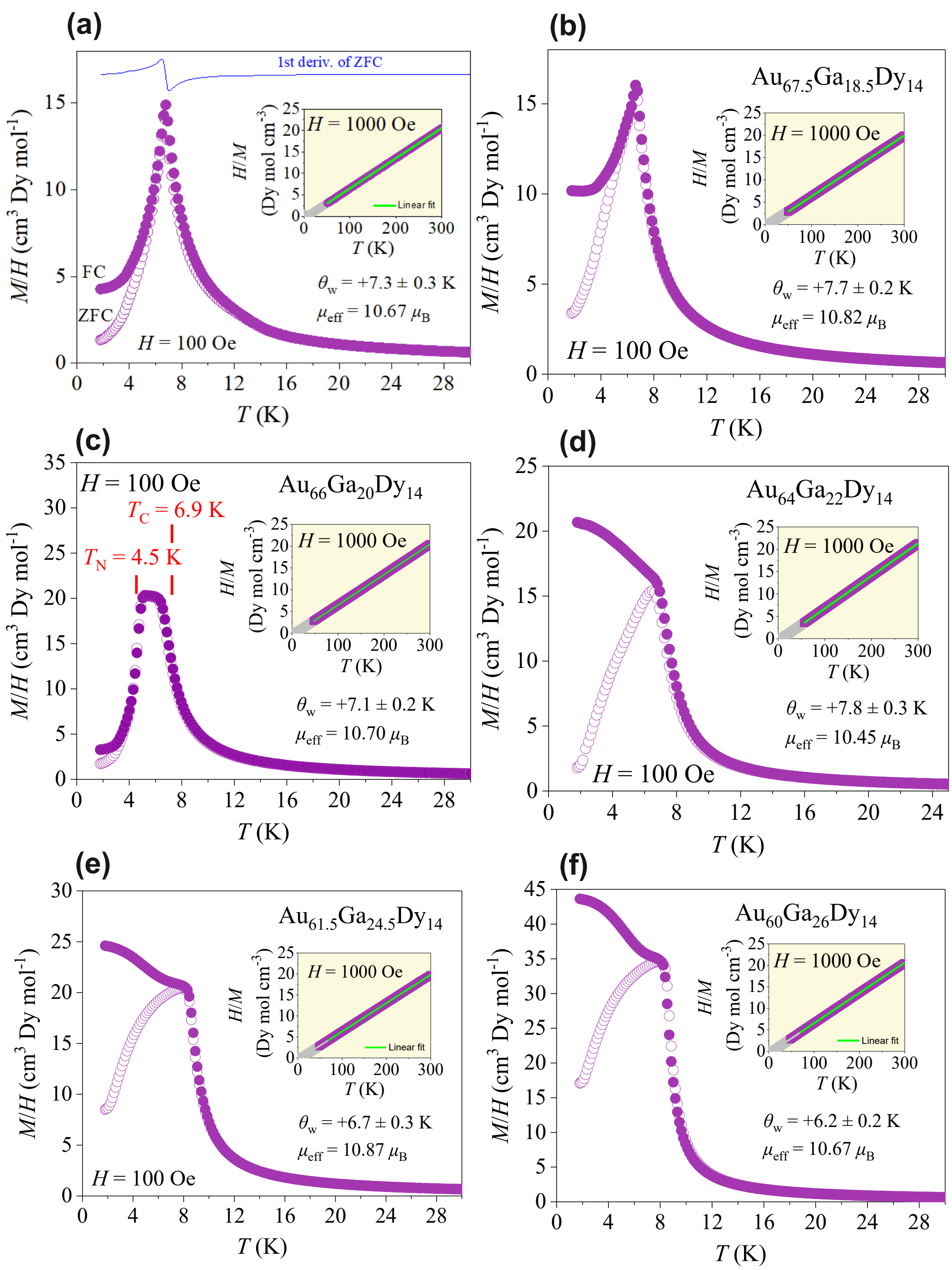

Fig. S5. Low-temperature magnetic susceptibility (M/H) of the (a) $Au_{68}Ga_{18}Dy_{14}$, (b) $Au_{67.5}Ga_{18.5}Dy_{14}$, (c) $Au_{66}Ga_{20}Dy_{14}$, (d) $Au_{64}Ga_{22}Dy_{14}$ (e) $Au_{61.5}Ga_{24.5}Dy_{14}$, (f) $Au_{60}Ga_{26}Dy_{14}$, (g) $Au_{57.5}Ga_{28.5}Dy_{14}$, (h) $Au_{55}Ga_{31}Dy_{14}$ and (i) $Au_{50}Ga_{36}Dy_{14}$ 1/1 ACs as a function of temperature under field cooled (FC) and zero-field-cooled (ZFC) modes and H = 100 Oe. The inset shows the corresponding inverse magnetic susceptibility (H/M) as a function of temperature under ZFC mode and H = 1000 Oe.

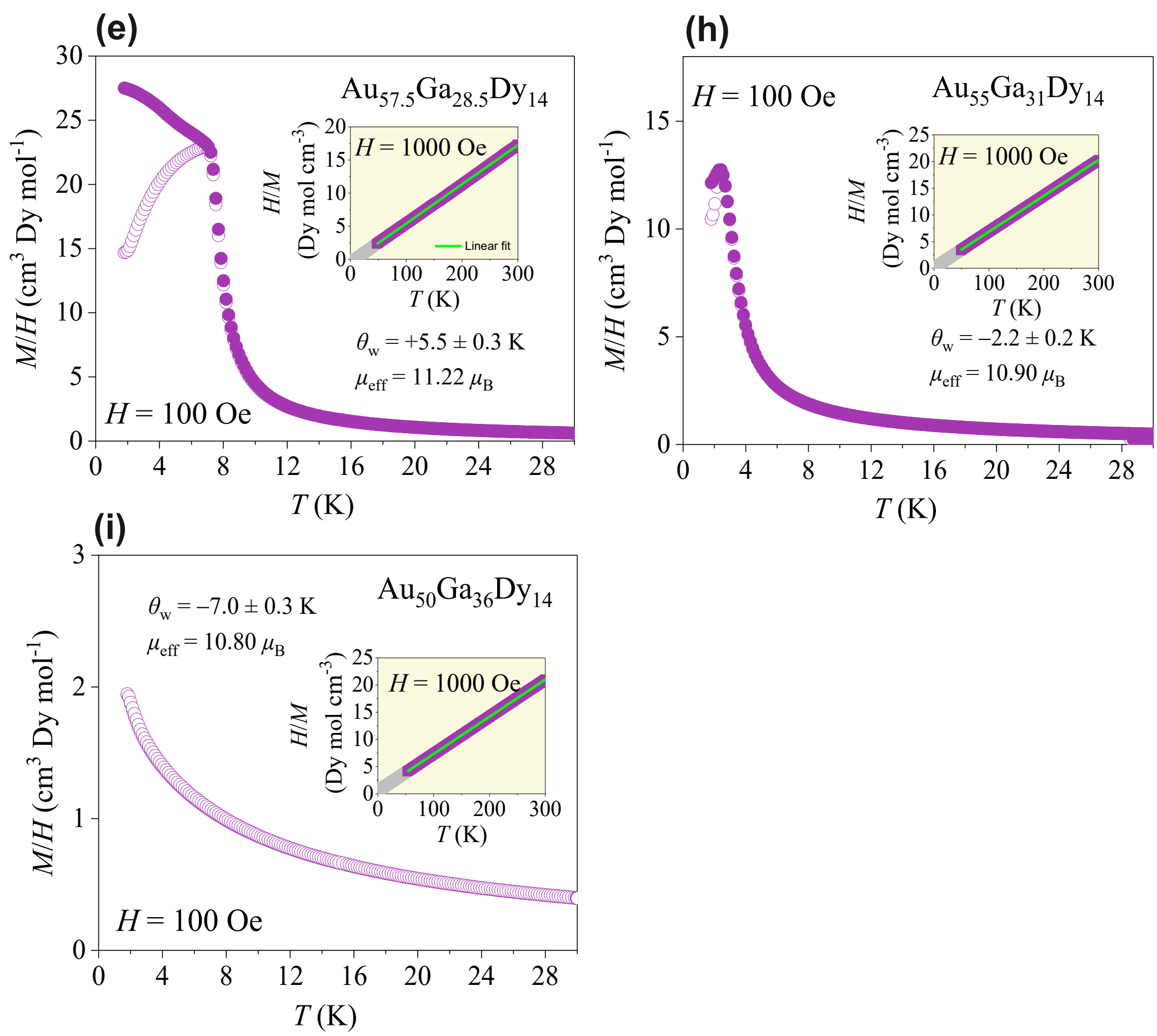

Fig. S5. Continued.

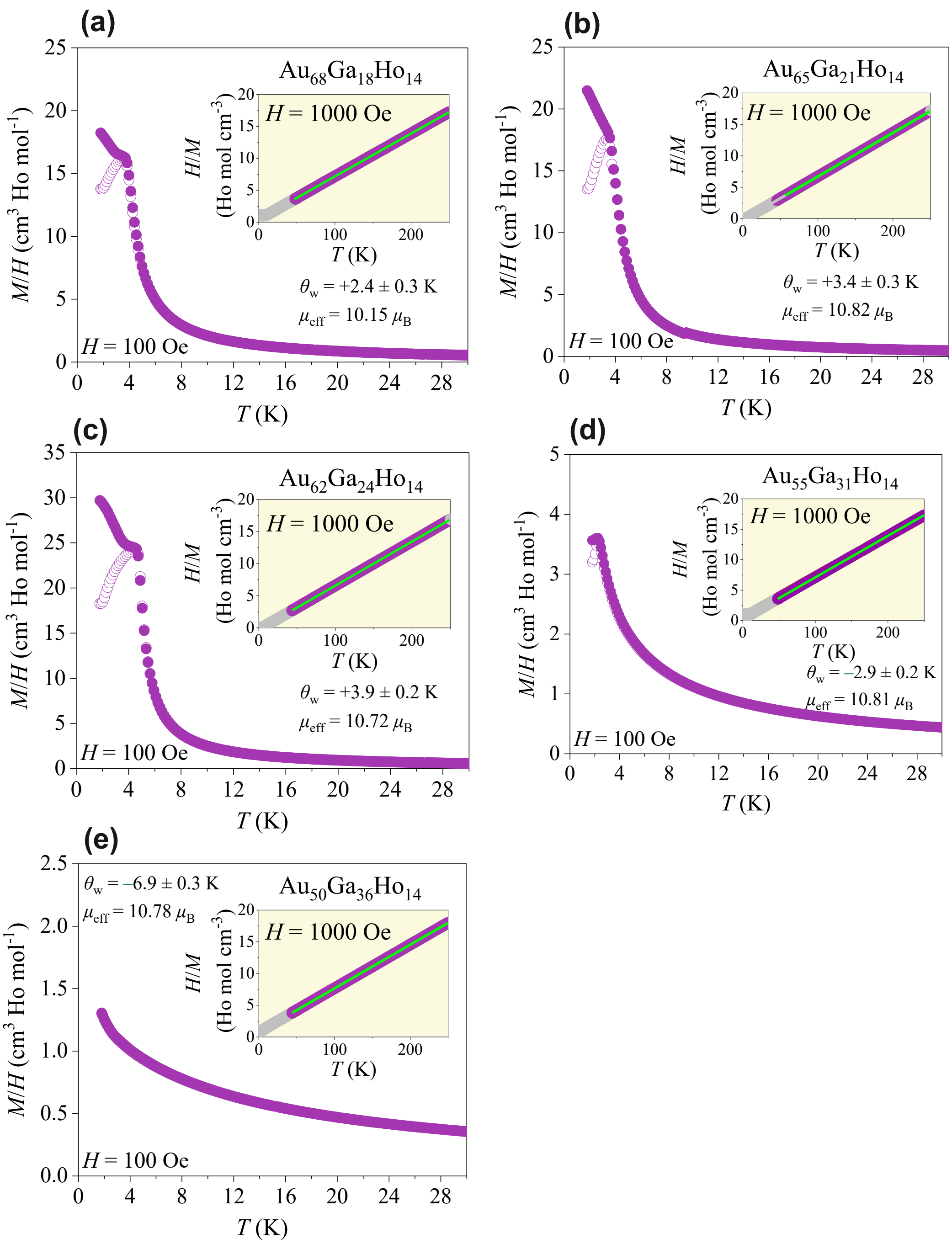

Fig. S6 Low-temperature *M/H* of the (a) $Au_{68}Ga_{18}Ho_{14}$, (b) $Au_{65}Ga_{21}Ho_{14}$, (c) $Au_{62}Ga_{24}Ho_{14}$, (d) $Au_{55}Ga_{31}Ho_{14}$ and (e) $Au_{50}Ga_{36}Ho_{14}$ 1/1 ACs as a function of temperature under FC and ZFC modes and H = 100 Oe. The inset shows the corresponding *H/M* as a function of temperature under ZFC mode and H = 1000 Oe.

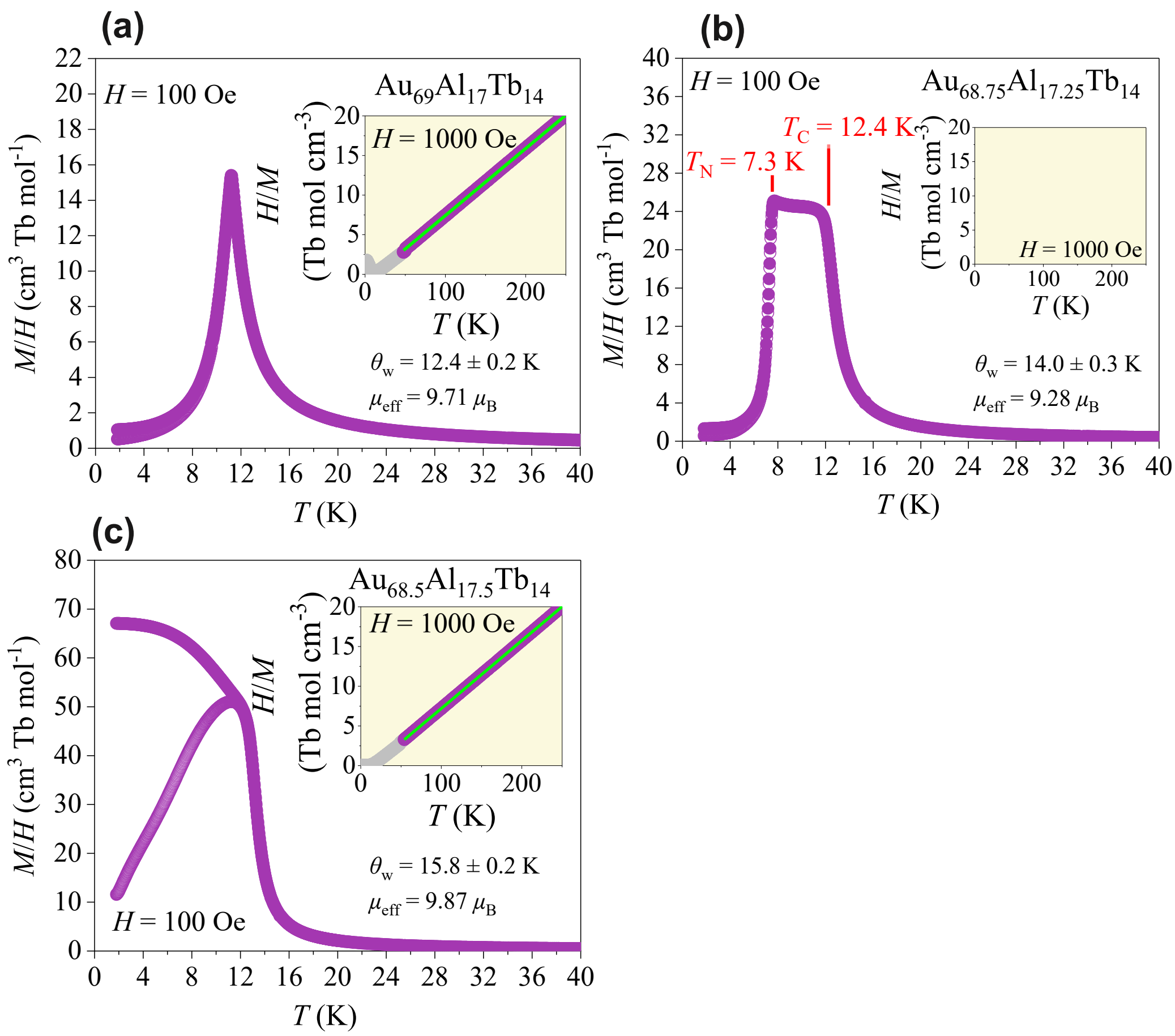

Fig. S7. Low-temperature *M/H* of the (a) $Au_{69}Al_{17}Tb_{14}$, (b) $Au_{68.75}Al_{17.25}Tb_{14}$, and (c) $Au_{68.5}Al_{17.5}Tb_{14}$ 1/1 ACs as a function of temperature under FC and ZFC modes and H = 100 Oe. The inset shows the corresponding *H/M* as a function of temperature under ZFC mode and H = 1000 Oe.

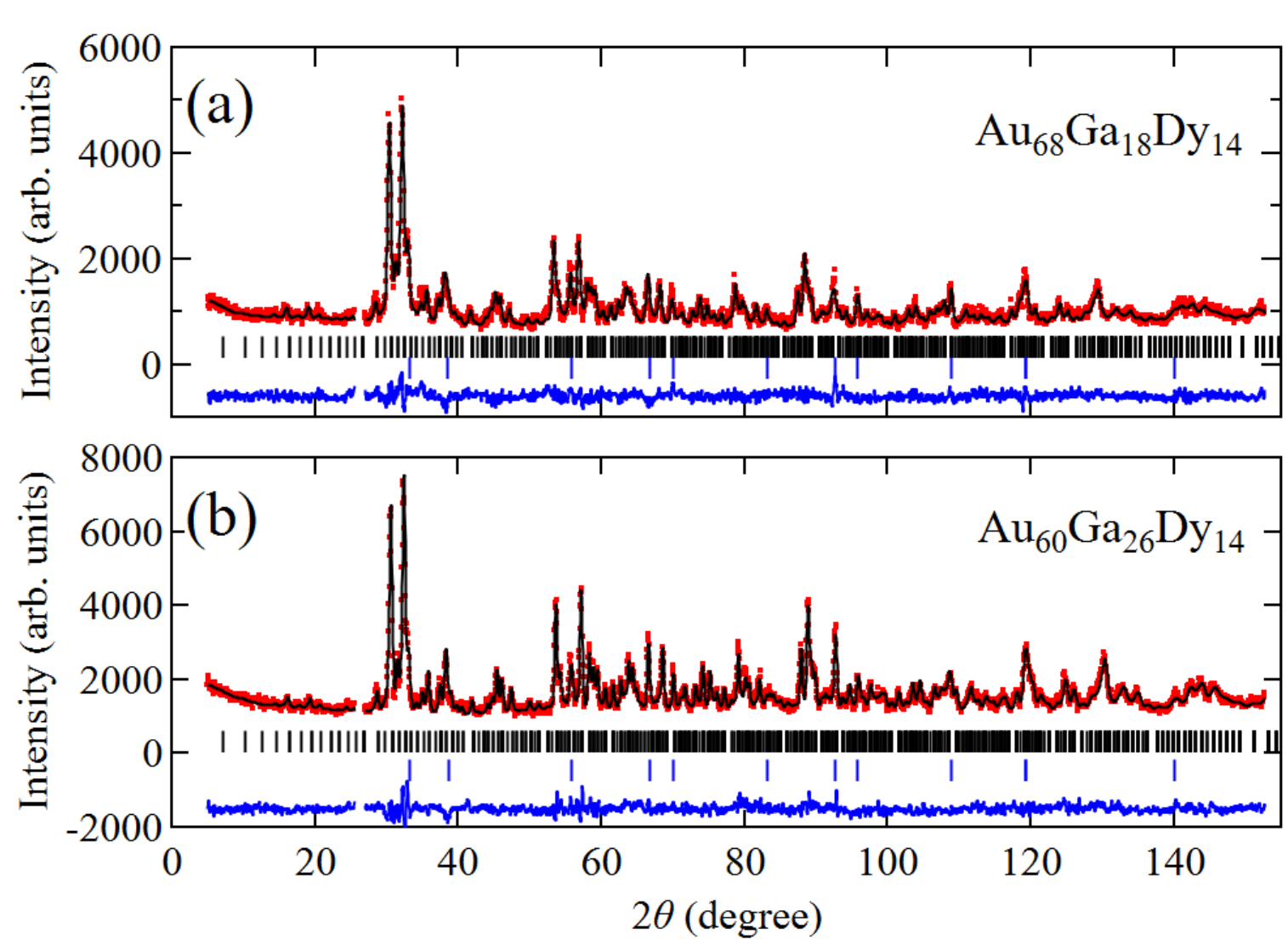

Fig. S8. Whole neutron diffraction patterns of (a) $Au_{68}Ga_{18}Dy_{14}$ and (b) $Au_{60}Ga_{26}Dy_{14}$ 1/1 ACs collected at 15 K.

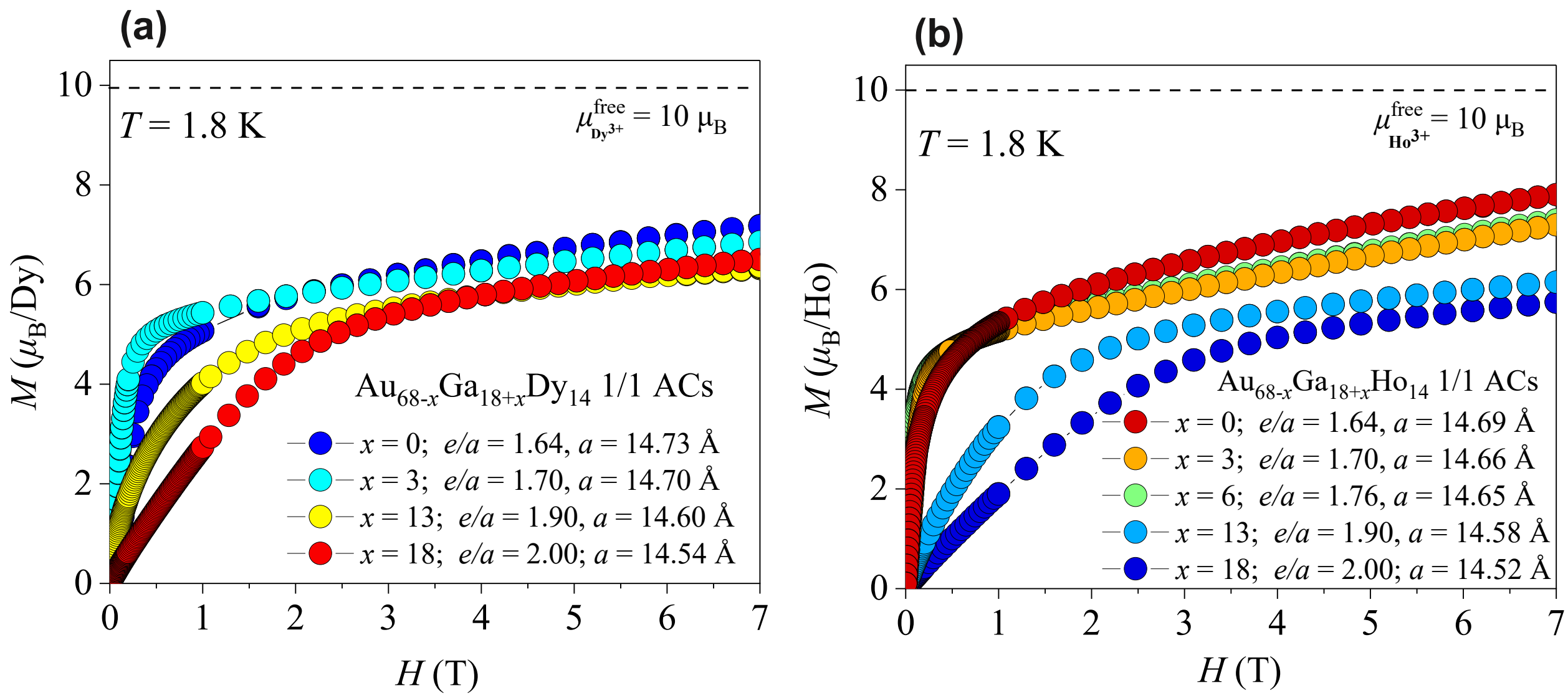

Fig. S9. Magnetic field dependence of magnetizations of (a) $Au_{68-x}Ga_{18+x}Dy_{14}$ (x = 0, 3, 13, and 18) and (b) $Au_{68-x}Ga_{18+x}Ho_{14}$ (x = 0, 3, 6, 13, 18) 1/1 ACs measured at 1.8 K up to μ_0H = 7 T.

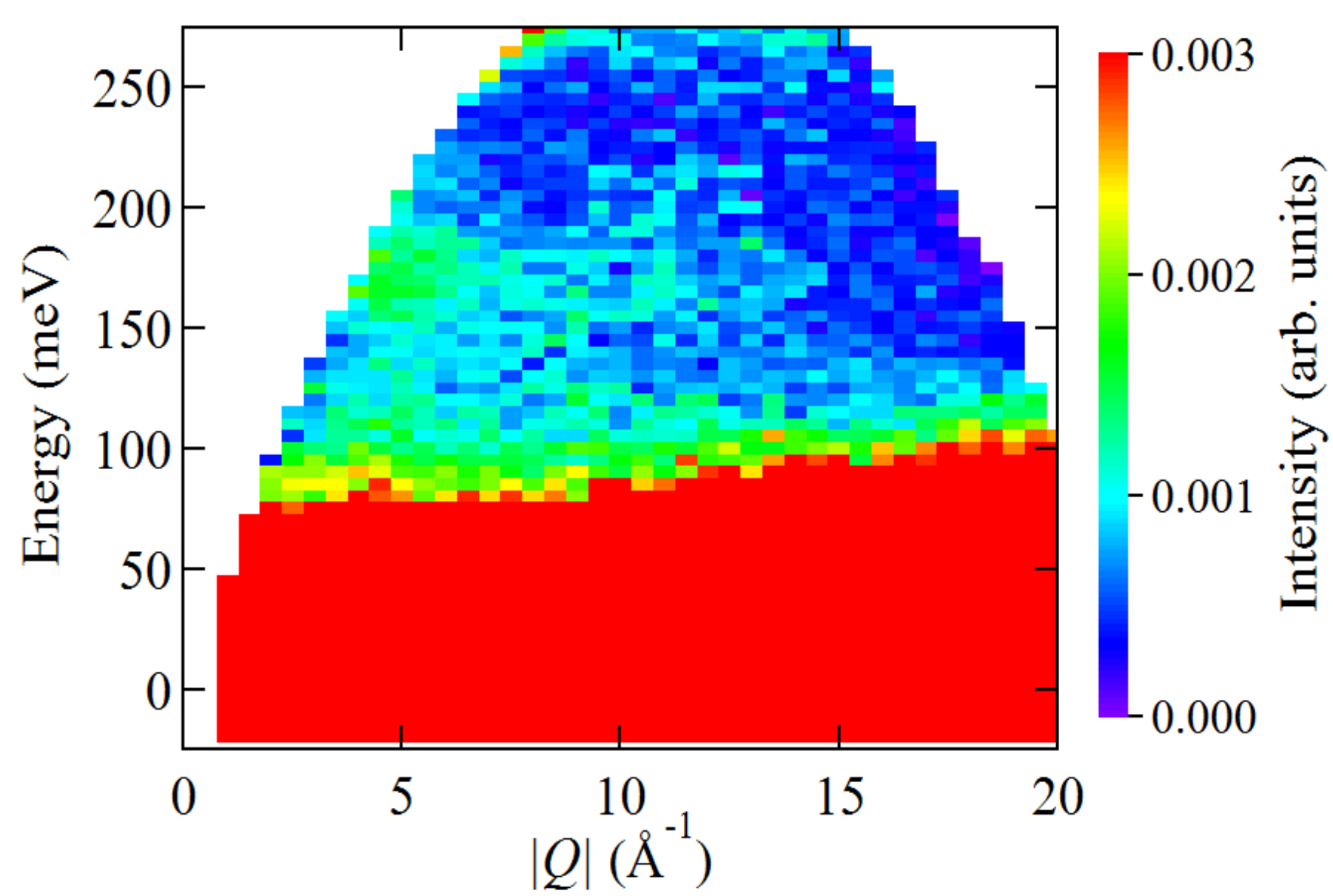

Fig. S10. Inelastic neutron scattering spectrum of $Au_{65}Ga_{21}Dy_{14}$ collected at 5 K. Incident neutron energy is set to 307 meV. A weak dispersionless mode was observed at 170 meV, which could be an intermultiplet transition of Dy^{3+}.

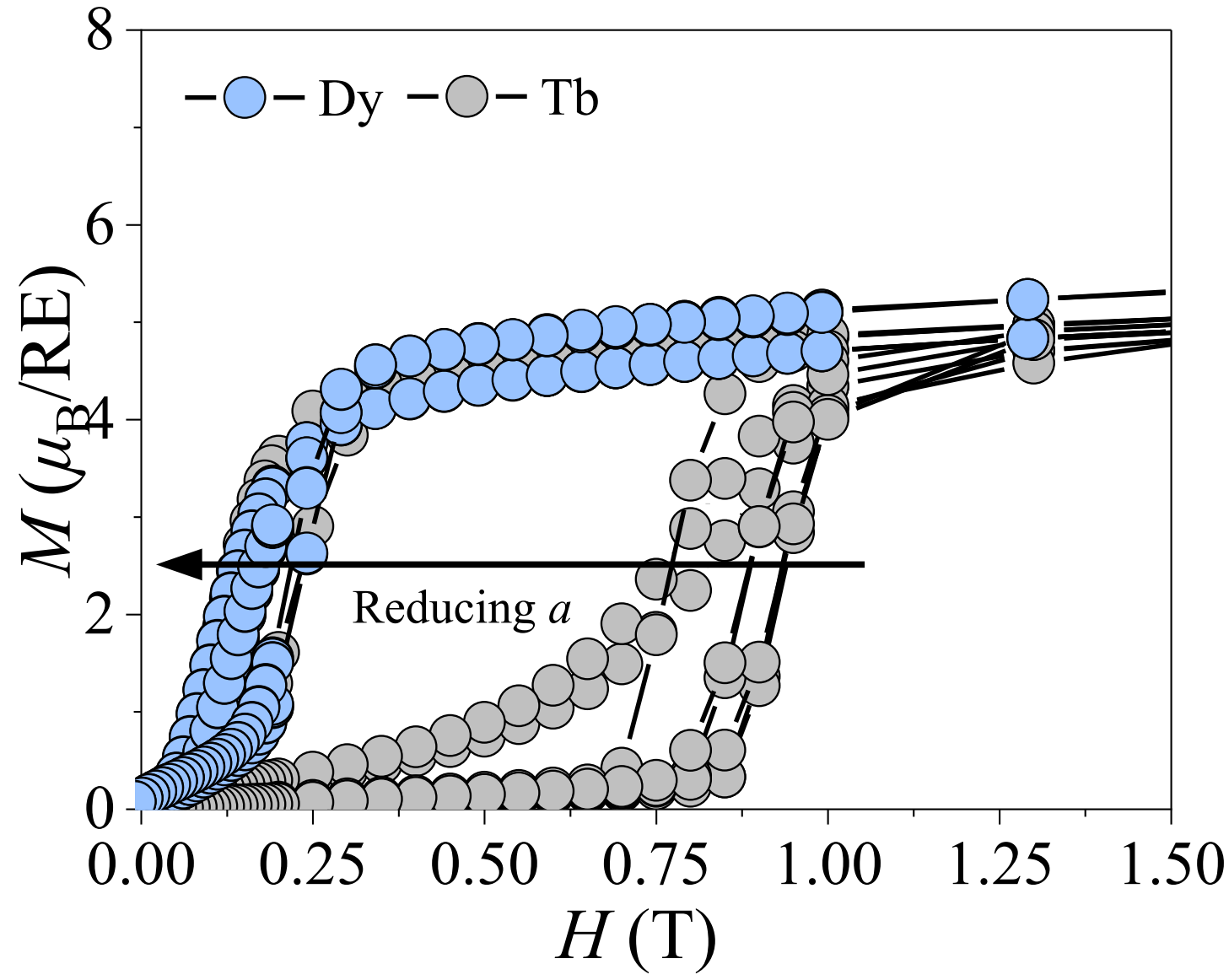

Fig. S11. Comparison of the field dependence of magnetization in AFM Au–Ga–Tb (grey) and AFM Au–Ga–Dy (blue) 1/1 ACs.

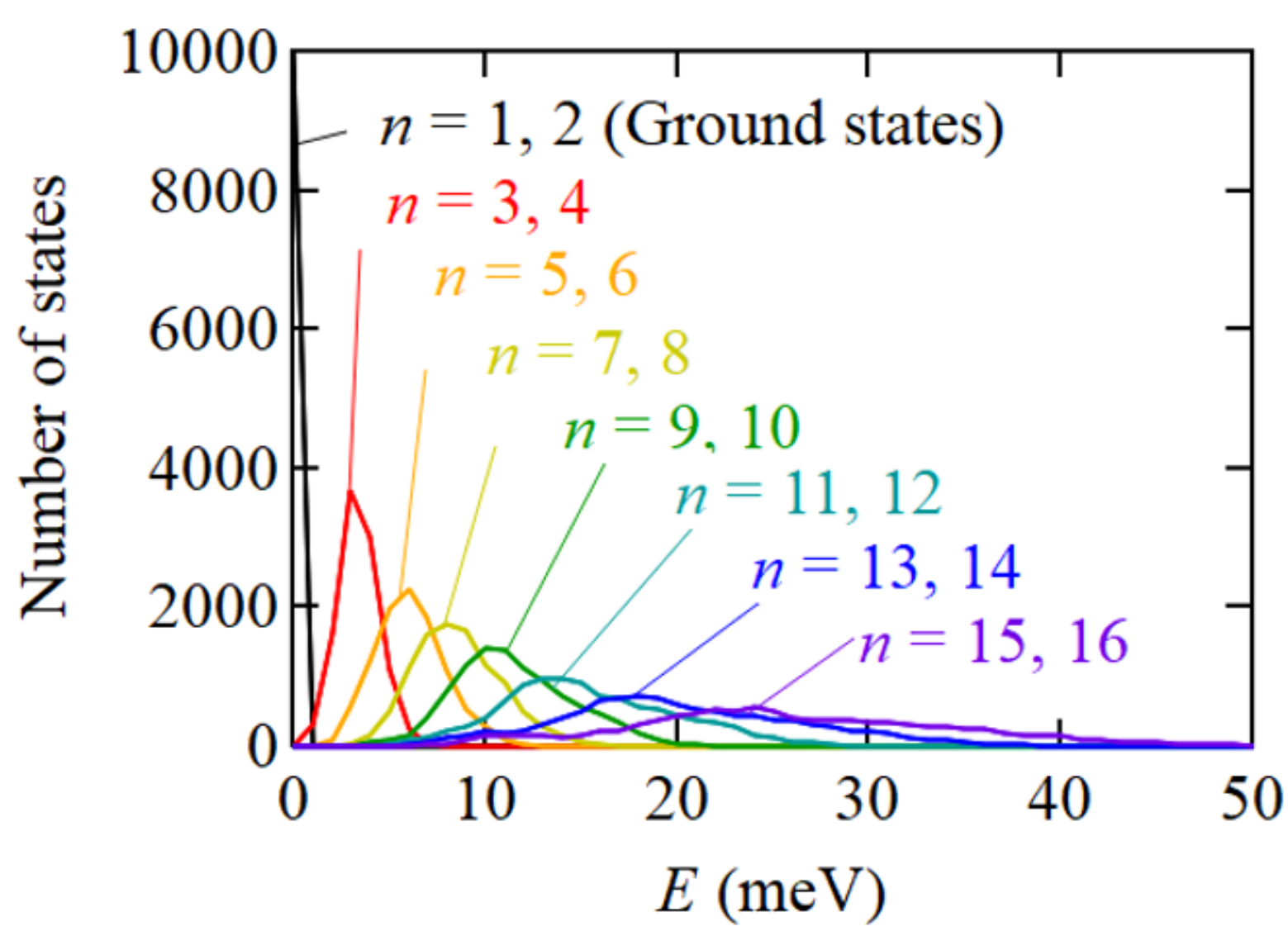

Fig. S12. Distribution of Dy crystal electric field (CEF) levels (n = 1, 2, …, 16) obtained from 5000 randomly generated atomic configurations. The lowest two levels correspond to the ground-state Kramers doublet. All CEF levels remain doubly degenerate.

Table S1. Crystallographic data and refinement parameters of the SCXRD structure analysis for $Au_{68}Ga_{18}Dy_{14}$ 1/1 AC

Crystallographic data and refinement parameters	
Space group	$Im\bar{3}$
a axis (Å)	14.7313(2)
Cell volume (Å^3)	3196.86(13)
Calculated density (g/cm³)	15.3212
Temperature of data collection (K)	298
h range	$-18 \le h \le 15$
k range	$-18 \le k \le 21$
l range	$-16 \le l \le 20$
F(000)	11950
Reflections collected	8370
Independent reflections	835
Data[a] / restraints / parameters	778/0/59
R indices for observed reflections (R, R_w)[b]	0.0346, 0.0575
Goodness of fit on *F*	1.206

[a] Reflections with $I > 2\sigma$ are used for the refinement.

[b] $R = \sum||F_{obs}|^2 - |F_{calc}|^2| / \sum|F_{obs}|^2$, $wR = \sum w(|F_{obs}|^2 - |F_{calc}|^2)/\sum w(|F_{obs}|^2$, $w = 1/[\sigma^2(|F_{obs}|^2) + (0.0164P)^2 + 263.5045P]$ where $P = (|F_{obs}|^2 + 2|F_{calc}|^2)/3$

Table S2. Crystallographic data and refinement parameters of the SCXRD structure analysis for $Au_{60}Ga_{26}Dy_{14}$ 1/1 AC

Crystallographic data and refinement parameters	
Space group	$Im\bar{3}$
a axis (Å)	14.6853(1)
Cell volume (Å^3)	3167.00(6)
Calculated density (g/cm³)	14.748
Temperature of data collection (K)	298
h range	$-20 \le h \le 20$
k range	$-20 \le k \le 19$
l range	$-20 \le l \le 20$
F(000)	11950
Reflections collected	8370
Independent reflections	835
Data[a] / restraints / parameters	778/0/59
R indices for observed reflections (R, R_w)[b]	0.0346, 0.0575
Goodness of fit on *F*	1.206

[a] Reflections with $I > 2\sigma$ are used for the refinement.

[b] $R = \sum||F_{obs}|^2 - |F_{calc}|^2| / \sum|F_{obs}|^2$, $wR = \sum w(|F_{obs}|^2 - |F_{calc}|^2)/\sum w(|F_{obs}|^2$, $w = 1/[\sigma^2(|F_{obs}|^2) + (0.0008\,P)^2 + 107.0057\,P]$ where $P = (|F_{obs}|^2 + 2|F_{calc}|^2)/3$

Table S3. Crystallographic data and refinement parameters of the SCXRD structure analysis for $Au_{62}Ga_{24}Ho_{14}$ 1/1 AC

Crystallographic data and refinement parameters	
Space group	$Im\overline{3}$
a axis (Å)	14.64840(10)
Cell volume (Å^3)	3143.19(6)
Calculated density (g/cm³)	14.943
Temperature of data collection (K)	299.0(5)
h range	$-25 \leq h \leq 24$
k range	$-25 \leq k \leq 24$
l range	$-24 \leq l \leq 25$
F(000)	11495
Reflections collected	20346
Independent reflections	1528
Data[a] / restraints / parameters	1427/2/101
R indices for observed reflections (R, R_w)[b]	0.0251, 0.0397
Goodness of fit on F	1.307

[a] Reflections with $I > 2\sigma$ are used for the refinement.

[b] $R = \sum||F_{obs}|^2 - |F_{calc}|^2| / \sum|F_{obs}|^2$, $wR = \sum w(|F_{obs}|^2 - |F_{calc}|^2)/\sum w(|F_{obs}|^2$, $w = 1/[\sigma^2(|F_{obs}|^2) + (0.0008\,P)^2 + 107.0057\,P]$ where $P = (|F_{obs}|^2 + 2|F_{calc}|^2)/3$

Table S4. Atomic coordinates, Wyckoff position, site occupation, and equivalent isotropic displacement parameter ($U_{eq.}$) of $Au_{68}Ga_{18}Dy_{14}$ 1/1 AC after refinement of the SCXRD data.

Unit/shell	Atom	Site	Wyck.	x	y	z	Occ.	U_{eq} (Å)^2
Tetrahedron	Au	Au09	24g	0	0.0777(6)	0.0735(7)	0.130(16)	0.067(5)
	Ga	Ga09	24g	0	0.0777(6)	0.0735(7)	0.163(16)	0.067(5)
	Au	Au10	48h	0.036(2)	0.045(2)	0.0762(18)	0.020(4)	0.014(11)
Dodecahedron	Au	Au03	16f	0.15074(4)	0.15074(4)	0.15074(4)	1	0.0240(2)
	Au	Au06	24g	0	0.2380(3)	0.0839(3)	0.54(3)	0.0228(9)
	Ga	Ga06	24g	0	0.2380(3)	0.0839(3)	0.43(3)	0.0228(9)
	Au	Au11	24g	0	0.261(6)	0.261(6)	0.261(6)	0.261(6)
Cubic interstices	Ga	Ga08	8c	0.25	0.25	0.25	0.985(17)	0.0245(13)
Icosahedron	Dy	Dy05	24g	0	0.18635(5)	0.30488(5)	1	0.01319(18)
Icosidodecahedron	Au	Au02	48h	0.10529(3)	0.34288(3)	0.20020(3)	1	0.01638(14)
	Au	Au04	12d	0.40539(7)	0	0	1	0.0264(3)
RTH	Au	Au01	24g	0	0.40399(4)	0.35280(4)	1	0.0140(16)
	Ga	Ga07	12e	0.19476(19)	0	0.5	1	0.0152(6)

Table S5. Atomic coordinates, Wyckoff position, site occupation, and equivalent isotropic displacement parameter ($U_{eq.}$) of $Au_{60}Ga_{26}Dy_{14}$ 1/1 AC after refinement of the SCXRD data.

Unit/shell	Atom	Site	Wyck.	*x*	*y*	*z*	Occ.	U_{eq} (Å)^2
Tetrahedron	Au	Au09	24*g*	0	0.0433(5)	0.0912(2)	0.152(4)	0.0299(15)
	Ga	Ga10	24*g*	0	0.0912(8)	0.0818(6)	0.182(4)	0.0275(18)
Dodecahedron	Au	Au03	16*f*	0.14930(2)	0.14930(2)	0.14930(2)	1	0.01705(10)
	Au	Au06	24*g*	0	0.2316(2)	0.0902(2)	0.342(4)	0.0248(6)
	Ga	Ga06	24*g*	0	0.2577(2)	0.0725(2)	0.658(4)	0.0120(6)
Cubic interstices	Ga	Ga08	8*c*	0.25	0.25	0.25	1	0.0183(4)
Icosahedron	Dy	Dy05	24*g*	0	0.18819(3)	0.30534(3)	1	0.01083(9)
Icosidodecahedron	Au	Au02	48*h*	0.10466(2)	0.34116(2)	0.20039(2)	1	0.01379(7)
	Au	Au04	12*d*	0	0.39823(15)	0	0.605(6)	0.0212(4)
	Ga	Ga04	12*d*	0	0.4234(5)	0	0.395(6)	0.020(2)
RTH	Au	Au01	24*g*	0	0.40399(2)	0.35622(2)	1	0.01209(8)
	Ga	Ga07	12*e*	0.19992(10)	0	0.5	1	0.0123(3)

Table S6. Atomic coordinates, Wyckoff position, site occupation, and equivalent isotropic displacement parameter ($U_{eq.}$) of $Au_{62}Ga_{24}Ho_{14}$ 1/1 AC after refinement of the SCXRD data.

Unit/shell	Atom	Site	Wyck.	*x*	*y*	*z*	Occ.	U_{eq} (Å)^2
Tetrahedron	Au	Au0C	24g	0.0795(15)	0.0479(19)	0	0.050(3)	0.069(6)
	Ga	Ga0B	24g	0.0468(7)	0.0867(7)	0	0.232(2)	0.0280(18)
	Au	Au0D	24g	0.0814(13)	0.0899(10)	0	0.051(3)	0.028(3)
Dodecahedron	Au	Au03	16f	0.14888(2)	0.14888(2)	0.14888(2)	0.926(4)	0.01846(13)
	Ga	Ga03	16f	0.169(3)	0.169(3)	0.169(3)	0.074(4)	0.058(12)
	Au	Au05	24*g*	0	0.2547(9)	0.0709(4)	0.140(3)	0.0162(11)
	Au	Au06	24g	0	0.2353(4)	0.0860(7)	0.276(3)	0.0200(10)
Cubic interstices	Ga	Ga09	8*c*	0.25	0.25	0.25	0.905(8)	0.0198(5)
Icosahedron	Ho	Ho0A	24g	0	0.18783(2)	0.30541(2)	1	0.01214(7)
Icosidodecahedron	Au	Au02	48h	0.10470(9)	0.34158(14)	0.20020(13)	0.86(3)	0.0147(2)
	Au	Au22	48h	0.1085(7)	0.3359(9)	0.1952(8)	0.14(3)	0.026(2)
	Au	Au04	12d	0.40093(12)	0	0	0.758(5)	0.0246(3)
RTH	Au	Au01	24g	0	0.40387(2)	0.35429(2)	1	0.01393(6)
	Ga	Ga08	12e	0.19726(9)	0	0.5	1	0.0141(2)
	Au	Au03	16f	0.14888(2)	0.14888(2)	0.14888(2)	0.926(4)	0.01846(13)

Table S7. Chemical composition, average electron concentration (*e/a*), lattice parameter, magnetic ground state, transition temperature (T_N, T_C and T_f), Curie-Weiss temperature θ_w and references used for constructing Fig. 2 of the main text.

Composition	*e/a*	Lattice parameter (Å)	T_f (K)	T_c (K)	T_N (K)	Θ_w (K)	μ_{eff} (μ_B)	$\mu_{calc.}$ (μ_B)	Magnetic ground state	Ref.
$Au_{70}Al_{16}Tb_{14}$	1.60	14.74	–	–	11.7	10.0	9.85	9.72	AFM	[3]
$Au_{72}Al_{14}Tb_{14}$	1.56	14.73	–	–	11.8	4.2	10.8	9.72	AFM	[4]
$Au_{69}Al_{17}Tb_{14}$	1.62	14.73	–	–	11.6	12.4	9.71	9.72	AFM	This work
$Au_{68.75}Al_{17.25}Tb_{14}$	1.625	14.72	–	12.4	7.3	14.0	9.28	9.72	FM/AFM	This work
$Au_{68.5}Al_{17.5}Tb_{14}$	1.63	14.71	–	13.4	–	15.8	9.87	9.72	FM	This work
$Au_{64}Al_{22}Tb_{14}$	1.72	14.68	–	15.0	–	18.7	9.92	9.72	FM	[5]
$Au_{53}Al_{33}Tb_{14}$	1.94	14.61	3.0	–	–	–9.9	10.41	9.72	SG	[6]
$Au_{70}Si_{17}Tb_{13}$	1.77	14.73†	–	11.6	–	12.4	9.66	9.72	FM	[7]
$Au_{70}Si_{16}Tb_{14}$	1.76	14.65	–	8.3	–	9.6	9.86	9.72	FM	[8]
$Au_{65.58}Si_{20.78}Tb_{13.64}$	1.89	14.64†	–	10.5	–	10.2	9.84	9.72	FM	[9]
$Au_{69.87}Si_{16.26}Tb_{13.87}$	1.76	14.62†	–	9	–	5.9	10.21	9.72	FM	[9]
$Au_{68}Ga_{18}Tb_{14}$	1.64	14.76	–	–	14.1	12.4	9.27	9.72	AFM	[10]
$Au_{67}Ga_{19}Tb_{14}$	1.66	14.76	–	–	13.8	13.2	9.95	9.72	AFM	[10]
$Au_{66}Ga_{20}Tb_{14}$	1.68	14.75	–	–	13.7	13.1	10.31	9.72	AFM	[10]
$Au_{65}Ga_{21}Tb_{14}$	1.70	14.73	–	–	13.1	12.1	10.39	9.72	AFM	[10]
$Au_{64}Ga_{22}Tb_{14}$	1.72	14.71	–	11.2	9.9	13.8	9.95	9.72	FM/AFM	[10]
$Au_{62}Ga_{24}Tb_{14}$	1.76	14.69	–	12.8	–	15.0	10.08	9.72	FM	[10]
$Au_{60}Ga_{26}Tb_{14}$	1.80	14.68	–	12.0	–	14.9	9.94	9.72	FM	[10]
$Au_{58}Ga_{28}Tb_{14}$	1.84	14.66	–	11.1	–	9.2	9.94	9.72	FM	[10]
$Au_{56}Ga_{30}Tb_{14}$	1.88	14.65	–	6.8	–	4.6	9.7	9.72	FM	[10]
$Au_{50}Ga_{36}Tb_{14}$	2.00	14.60	2.8	–	–	–17.3	9.8	9.72	SG	[10]
$Au_{45}Ga_{41}Tb_{14}$	2.10	14.52	2.6	–	–	–19.7	10.7	9.72	SG	[10]
$Au_{41}Ga_{45}Tb_{14}$	2.18	14.50	2.6	–	–	–20.2	10.0	9.72	SG	[10]
$Au_{68}Ga_{18}Dy_{14}$	1.64	14.73	–	–	6.71	7.3	10.67	10.63	AFM	This work
$Au_{67.5}Ga_{18.5}Dy_{14}$	1.65	14.73	–	–	6.91	7.7	10.82	10.63	AFM	This work
$Au_{66}Ga_{20}Dy_{14}$	1.68	14.72	–	6.90	4.52	7.1	10.70	10.63	FM/AFM	This work
$Au_{65}Ga_{21}Dy_{14}$	1.70	14.70	–	9.2	–	7.8	10.67	10.63	FM	This work
$Au_{61.5}Ga_{24.5}Dy_{14}$	1.77	14.69	–	8.7	–	6.7	10.87	10.63	FM	This work
$Au_{60}Ga_{26}Dy_{14}$	1.80	14.68	–	8.6	–	6.2	10.67	10.63	FM	This work
$Au_{57.5}Ga_{28.5}Dy_{14}$	1.85	14.64		6.8	–	5.5	11.22	10.63	FM	This work
$Au_{55}Ga_{31}Dy_{14}$	1.90	14.60	3.1	–	–	–2.2	10.90	10.63	SG	This work
$Au_{50}Ga_{36}Dy_{14}$	2.00	14.54	< 2	–	–	–7.0	10.80	10.63	SG	This work
$Au_{68}Ga_{18}Ho_{14}$	1.64	14.69	–	3.8	–	2.4	10.15	10.58	FM	This work
$Au_{65}Ga_{21}Ho_{14}$	1.70	14.66	–	3.9	–	3.4	10.82	10.58	FM	This work
$Au_{62}Ga_{24}Ho_{14}$	1.76	14.65	–	5.0	–	3.9	10.72	10.58	FM	This work
$Au_{55}Ga_{31}Ho_{14}$	1.90	14.58	2.3	–	–	–2.9	10.81	10.58	SG	This work
$Au_{50}Ga_{36}Ho_{14}$	2.00	14.52	< 2	–	–	–6.9	1078	10.58	SG	This work
$Au_{61.1}Al_{25.3}Ho_{13.6}$	1.78	14.63†		6		3.7	10.75	10.58	FM	[1]
$Au_{68.60}Si_{16.71}Ho_{14.70}$	1.80	14.62†		3		2.9	10.81	10.58	FM	[9]

†These lattice parameters were determined at low temperature; the corresponding room-temperature values may be slightly larger.

Table S8. Refined crystallographic data of the powder neutron diffraction experiments. $C_{\mathrm{AFM},1}$ and $C_{\mathrm{AFM},2}$ correspond to the two coefficients of the IR2-1 and IR2-2 in Table S8, which are equivalent to the components of the magnetic moments along the *y*- and *z*- axis at the Dy atom present at the fractional coordinate (0, 0.1842, 0.3051) in $Au_{68}Ga_{18}Dy_{14}$, respectively. On the other hand, $C_{\mathrm{FM},1}$, $C_{\mathrm{FM},2}$, and $C_{\mathrm{FM},3}$ correspond to the coefficients $C_{\mathrm{FM},1} \equiv C_{\mathrm{IR8-1:3}} = C_{\mathrm{IR8-3:2}} = C_{\mathrm{IR8-5:1}}$, $C_{\mathrm{FM},2} \equiv C_{\mathrm{IR8-2:1}} = C_{\mathrm{IR8-4:1}} = C_{\mathrm{IR8-5:2}}$, and $C_{\mathrm{FM},3} \equiv C_{\mathrm{IR8-2:2}} = C_{\mathrm{IR8-4:2}} = C_{\mathrm{IR8-5:3}}$, which are equivalent to the components of the magnetic moments along the *x*-, *y*-, and *z*-axis at the Dy atom present at the fractional coordinate (0, 0.1879, 0.3063) in $Au_{60}Ga_{26}Dy_{14}$.

	$Au_{68}Ga_{14}Dy_{14}$		$Au_{60}Ga_{22}Dy_{14}$	
	2 K	15 K	2 K	15 K
a (Å)	14.6871(3)	14.6865(4)	14.6366(3)	14.6334(3)
V (Å^3)	3168.19(20)	3167.75(24)	3135.58(17)	3133.56(22)
$C_{\mathrm{AFM},1}$ (μ_B)	5.35(12)	–	–	–
$C_{\mathrm{AFM},2}$ (μ_B)	−1.59(16)	–	–	–
$C_{\mathrm{FM},1}$ (μ_B)	–	–	2.52(29)	–
$C_{\mathrm{FM},2}$ (μ_B)	–	–	4.52(17)	–
$C_{\mathrm{FM},3}$ (μ_B)	–	–	−0.92(35)	–
Calculated density (g/cm^3)	15.46	15.46	14.90	14.91
No. of parameters	39	37	39	36
2θ range used for refinement (°)	5.0–25.5, 27.0–152.7			
No. of nuclear reflections	928	928	920	920
No. of magnetic reflections	9996	–	9858	–
R_p (%)	18.40	19.70	18.30	19.30
R_{wp} (%)	19.80	20.40	18.90	19.30
R_e (%)	12.54	13.32	10.84	11.46
χ^2	2.50	2.34	3.04	2.83
R_{mag}	10.4	–	9.53	–

Table S9. Structure parameters of $Au_{68}Ga_{18}Dy_{14}$ 1/1 AC at 15 K determined from the PND experiment. The space group is $Im\overline{3}$, and the lattice parameter is a = 14.6871(3) Å. The atomic coordination is represented by fractional coordinates. The equivalent isotropic displacement parameters B_{iso} are listed in a unit of Å^2. Atomic positions of the atoms in tetrahedron units and occupancies of all the atoms are fixed to the values determined from the SXRD experiments.

atom	site	x	y	z	B_{iso}	occupancy
Au01	24*g*	0	0.4004(7)	0.3565(7)	0.65(6)	1
Au02	48*h*	0.1029(5)	0.3406(4)	0.2000(5)	0.65(6)	1
Au03	16*f*	0.1478(5)	0.1478(5)	0.1478(5)	0.65(6)	1
Au04	12*d*	0.4023(10)	0	0	0.65(6)	1
Dy05	24*g*	0	0.1845(3)	0.3057(3)	0.60(6)	1
Au06	24*g*	0	0.2385(9)	0.0852(9)	1.46(16)	0.54
Ga06	24*g*	0	0.2385(9)	0.0852(9)	1.46(16)	0.43
Ga07	12*e*	0.1986(10)	0	0.5	0.58(14)	1
Ga08	8*c*	0.25	0.25	0.25	0.58(14)	0.985
Au09	24*g*	0	0.0777	0.0735	1.46(16)	0.130
Ga09	24*g*	0	0.0777	0.0735	1.46(16)	0.163
Au10	48*h*	0.0360	0.0450	0.0762	0.65(6)	0.020
Au11	24*g*	0	0.2610	0.1050	0.65(6)	0.034

Table S10. Structure parameters of $Au_{68}Ga_{18}Dy_{14}$ 1/1 AC at 2 K determined from the PND experiment. The space group is $Im\bar{3}$, and the lattice parameter is a = 14.6871(3) Å. The atomic coordination is represented by fractional coordinates. The equivalent isotropic displacement parameters B_{iso} are listed in a unit of Å^2. Atomic positions of the atoms in tetrahedron units and occupancies of all the atoms are fixed to the values determined from the SXRD experiments.

atom	site	x	y	z	B_{iso}	occupancy
Au01	24*g*	0	0.3988(7)	0.3550(7)	0.57(5)	1
Au02	48*h*	0.1049(5)	0.3415(4)	0.2007(5)	0.57(5)	1
Au03	16*f*	0.1485(5)	0.1485(5)	0.1485(5)	0.57(5)	1
Au04	12*d*	0.4020(10)	0	0	0.57(5)	1
Dy05	24*g*	0	0.1842(3)	0.3051(3)	0.59(6)	1
Au06	24*g*	0	0.2388(10)	0.0844(10)	1.90(19)	0.54
Ga06	24*g*	0	0.2388(10)	0.0844(10)	1.90(19)	0.43
Ga07	12*e*	0.1996(12)	0	0.5	1.08(16)	1
Ga08	8*c*	0.25	0.25	0.25	1.08(16)	0.985
Au09	24*g*	0	0.0777	0.0735	1.90(19)	0.130
Ga09	24*g*	0	0.0777	0.0735	1.90(19)	0.163
Au10	48*h*	0.0360	0.0450	0.0762	0.57(5)	0.020
Au11	24*g*	0	0.2610	0.1050	0.57(5)	0.034

Table S11 Irreducible representations for the Dy site in $Au_{68}Ga_{18}Dy_{14}$ and $Au_{60}Ga_{26}Dy_{14}$. List of basis vectors of all the irreducible representations for the Dy site in with the space group *Im*-3 and the magnetic modulation vector of k = (1,1,1) ($Au_{68}Ga_{18}Dy_{14}$). The list can be also applied to the irreducible representations for the Dy site with the space group *Im*-3 and the magnetic modulation vector of k = (0,0,0) ($Au_{60}Ga_{26}Dy_{14}$). The atomic coordinates of the 12 Dy sites forming a nearly icosahedral cluster on the origin are listed in Table S11. The rest of the atoms, which form a cluster on the (1/2, 1/2, 1/2) position, are induced by body-centering symmetry. The number in parentheses represents a dimension of each IR. ϵ represents 1+i $\sqrt{3}/2$.

IRs	Dy1	Dy2	Dy3	Dy4	Dy5	Dy6	Dy7	Dy8	Dy9	Dy10	Dy11	Dy12
IR1 (1)	1 0 0	−1 0 0	−1 0 0	1 0 0	0 1 0	0 −1 0	0 −1 0	0 1 0	0 0 1	0 0 −1	0 0 −1	0 0 1
IR2-1 (1)	0 1 0	0 −1 0	0 1 0	0 −1 0	0 0 1	0 0 −1	0 0 1	0 0 −1	1 0 0	−1 0 0	1 0 0	−1 0 0
IR2-2 (1)	0 0 1	0 0 1	0 0 −1	0 0 −1	1 0 0	1 0 0	−1 0 0	−1 0 0	0 1 0	0 1 0	0 −1 0	0 −1 0
IR3 (1)	1 0 0	−1 0 0	−1 0 0	1 0 0	0 $-\epsilon$ 0	0 ϵ 0	0 ϵ 0	0 $-\epsilon$ 0	0 0 $-\epsilon^*$	0 0 ϵ^*	0 0 ϵ^*	0 0 $-\epsilon^*$
IR4-1 (1)	0 1 0	0 −1 0	0 1 0	0 −1 0	0 0 -ϵ	0 0 ϵ	0 0 $-\epsilon$	0 0 ϵ	$-\epsilon^*$ 0 0	ϵ^* 0 0	$-\epsilon^*$ 0 0	ϵ^* 0 0
IR4-2 (1)	0 0 1	0 0 1	0 0 −1	0 0 −1	$-\epsilon$ 0 0	$-\epsilon$ 0 0	ϵ 0 0	ϵ 0 0	0 $-\epsilon^*$ 0	0 $-\epsilon^*$ 0	0 ϵ^* 0	0 ϵ^* 0
IR5 (1)	1 0 0	−1 0 0	−1 0 0	1 0 0	0 $-\epsilon^*$ 0	0 ϵ^* 0	0 ϵ^* 0	0 $-\epsilon^*$ 0	0 0 $-\epsilon$	0 0 ϵ	0 0 ϵ	0 0 $-\epsilon$
IR6-1 (1)	0 1 0	0 −1 0	0 1 0	0 −1 0	0 0 $-\epsilon^*$	0 0 ϵ^*	0 0 $-\epsilon^*$	0 0 ϵ^*	$-\epsilon$ 0 0	ϵ 0 0	$-\epsilon$ 0 0	ϵ 0 0
IR6-1 (1)	0 0 1	0 0 1	0 0 −1	0 0 −1	$-\epsilon$ 0 0	-ϵ 0 0	ϵ^* 0 0	ϵ^* 0 0	0 $-\epsilon$ 0	0 $-\epsilon$ 0	0 ϵ 0	0 ϵ 0
IR7-1:1 (3)	1 0 0	−1 0 0	1 0 0	−1 0 0	0 0 0	0 0 0	0 0 0	0 0 0	0 0 0	0 0 0	0 0 0	0 0 0
IR7-1:2	0 0 0	0 0 0	0 0 0	0 0 0	0 0 0	0 0 0	0 0 0	0 0 0	1 0 0	1 0 0	−1 0 0	−1 0 0
IR7-1:3	0 0 0	0 0 0	0 0 0	0 0 0	0 0 0	0 0 0	0 0 0	0 0 0	0 1 0	0 −1 0	0 1 0	0 −1 0
IR7-2:1 (3)	0 0 0	0 0 0	0 0 0	0 0 0	0 1 0	0 1 0	0 −1 0	0 −1 0	0 0 0	0 0 0	0 0 0	0 0 0
IR7-2:2	0 0 0	0 0 0	0 0 0	0 0 0	0 1 0	0 −1 0	0 1 0	0 −1 0	0 0 0	0 0 0	0 0 0	0 0 0
IR7-2:3	0 1 0	0 1 0	0 −1 0	0 −1 0	0 0 0	0 0 0	0 0 0	0 0 0	0 0 0	0 0 0	0 0 0	0 0 0
IR7-3:1 (3)	0 0 1	0 0 −1	0 0 1	0 0 −1	0 0 0	0 0 0	0 0 0	0 0 0	0 0 0	0 0 0	0 0 0	0 0 0
IR7-3:2	0 0 0	0 0 0	0 0 0	0 0 0	0 0 0	0 0 0	0 0 0	0 0 0	0 0 1	0 0 1	0 0 −1	0 0 −1
IR7-3:3	0 0 0	0 0 0	0 0 0	0 0 0	0 0 0	0 0 0	0 0 0	0 0 0	0 0 1	0 0 −1	0 0 1	0 0 −1
IR7-4:1 (3)	0 0 0	0 0 0	0 0 0	0 0 0	0 0 1	0 0 1	0 0 −1	0 0 −1	0 0 0	0 0 0	0 0 0	0 0 0
IR7-4:2	0 0 0	0 0 0	0 0 0	0 0 0	1 0 0	−1 0 0	1 0 0	−1 0 0	0 0 0	0 0 0	0 0 0	0 0 0
IR7-4:3	1 0 0	1 0 0	−1 0 0	−1 0 0	0 0 0	0 0 0	0 0 0	0 0 0	0 0 0	0 0 0	0 0 0	0 0 0
IR8-1:1 (3)	0 1 0	0 −1 0	0 −1 0	0 1 0	0 0 0	0 0 0	0 0 0	0 0 0	0 0 0	0 0 0	0 0 0	0 0 0
IR8-1:2	0 0 1	0 0 1	0 0 1	0 0 1	0 0 0	0 0 0	0 0 0	0 0 0	0 0 0	0 0 0	0 0 0	0 0 0
IR8-1:3	0 0 0	0 0 0	0 0 0	0 0 0	0 0 0	0 0 0	0 0 0	0 0 0	0 0 1	0 0 1	0 0 1	0 0 1
IR8-2:1 (3)	0 0 0	0 0 0	0 0 0	0 0 0	0 0 1	0 0 1	0 0 1	0 0 1	0 0 0	0 0 0	0 0 0	0 0 0
IR8-2:2	0 0 0	0 0 0	0 0 0	0 0 0	1 0 0	−1 0 0	−1 0 0	1 0 0	0 0 0	0 0 0	0 0 0	0 0 0
IR8-2:3	0 0 0	0 0 0	0 0 0	0 0 0	0 0 1	0 0 −1	0 0 −1	0 0 1	0 0 0	0 0 0	0 0 0	0 0 0
IR8-3:1 (3)	0 0 0	0 0 0	0 0 0	0 0 0	1 0 0	1 0 0	1 0 0	1 0 0	0 0 0	0 0 0	0 0 0	0 0 0
IR8-3:2	1 0 0	1 0 0	1 0 0	1 0 0	0 0 0	0 0 0	0 0 0	0 0 0	0 0 0	0 0 0	0 0 0	0 0 0
IR8-3:3	0 0 0	0 0 0	0 0 0	0 0 0	0 0 0	0 0 0	0 0 0	0 0 0	1 0 0	1 0 0	1 0 0	1 0 0
IR8-4:1 (3)	0 0 0	0 0 0	0 0 0	0 0 0	0 0 0	0 0 0	0 0 0	0 0 0	0 1 0	0 −1 0	0 −1 0	0 1 0
IR8-4:2	0 0 0	0 0 0	0 0 0	0 0 0	0 0 0	0 0 0	0 0 0	0 0 0	1 0 0	−1 0 0	−1 0 0	1 0 0
IR8-4:3	0 0 0	0 0 0	0 0 0	0 0 0	0 0 0	0 0 0	0 0 0	0 0 0	0 1 0	0 1 0	0 1 0	0 1 0
IR8-5:1 (3)	0 0 0	0 0 0	0 0 0	0 0 0	0 1 0	0 1 0	0 1 0	0 1 0	0 0 0	0 0 0	0 0 0	0 0 0
IR8-5:2	0 1 0	0 1 0	0 1 0	0 1 0	0 0 0	0 0 0	0 0 0	0 0 0	0 0 0	0 0 0	0 0 0	0 0 0
IR8-5:3	0 0 1	0 0 −1	0 0 −1	0 0 1	0 0 0	0 0 0	0 0 0	0 0 0	0 0 0	0 0 0	0 0 0	0 0 0

Table S12. List of the atomic coordinates of the Dy1-12 sites in $Au_{68}Ga_{14}Dy_{14}$ and $Au_{60}Ga_{22}Dy_{14}$. y and z represent the fractional coordinate 0.1842 and 0.3051 ($Au_{68}Ga_{14}Dy_{14}$), or 0.1879 and 0.3063 ($Au_{60}Ga_{26}Dy_{14}$), respectively. The rest of the atoms are induced by body-centering symmetry, +(1/2, 1/2, 1/2).

IRs		IRs		IRs	
Dy1	0, y, z	Dy5	z, 0, y	Dy9	y, z, 0
Dy2	0, $-y$+1 ,z	Dy6	z, 0, $-y$+1	Dy10	$-y$+1, z, 0
Dy3	0, y, $-z$+1	Dy7	$-z$+1, 0, y	Dy11	y, $-z$+1, 0
Dy4	0, $-y$+1, $-z$+1	Dy8	$-z$+1, 0, $-y$+1	Dy12	$-y$+1, $-z$+1, 0

Table S13. Structure parameters of $Au_{60}Ga_{26}Dy_{14}$ 1/1 AC at 15 K determined from the PND experiment. The space group is $Im\bar{3}$, and the lattice parameter is a = 14.6334(3) Å. The atomic coordination is represented by fractional coordinates. The equivalent isotropic displacement parameters B_{iso} are listed in a unit of Å^2. Atomic positions of the atoms in tetrahedron units and occupancies of all the atoms are fixed to the values determined from the SXRD experiments.

atom	site	x	y	z	B_{iso}	occupancy
Au01	24*g*	0	0.4047(5)	0.3576(5)	0.15(4)	1
Au02	48*h*	0.1035(4)	0.3411(3)	0.1989(3)	0.15(4)	1
Au03	16*f*	0.1484(4)	0.1484(4)	0.1484(4)	0.15(4)	1
Au04	12*d*	0	0.4092(9)	0	0.79(14)	0.605
Ga04	12*d*	0	0.4092(9)	0	0.79(14)	0.395
Dy05	24*g*	0	0.1883(3)	0.3068(2)	0.20	1
Ga06	24*g*	0	0.2349(9)	0.0936(9)	0.79(14)	0.658
Au06	24*g*	0	0.2560(9)	0.0622(9)	0.79(14)	0.342
Ga07	12*e*	0.1991(8)	0	0.5	0.19(9)	1
Ga08	8*c*	0.25	0.25	0.25	0.19(9)	1
Au09	24*g*	0	0.0433	0.0912	0.15(4)	0.152
Ga10	24*g*	0	0.0912	0.0818	0.19(9)	0.182

Table S14. Structure parameters of $Au_{60}Ga_{26}Dy_{14}$ 1/1 AC at 2 K determined from the PND experiment. The space group is $Im\bar{3}$, and the lattice parameter is a = 14.6366(3) Å. The atomic coordination is represented by fractional coordinates. The equivalent isotropic displacement parameters B_{iso} are listed in a unit of Å^2. Atomic positions of the atoms in tetrahedron units and occupancies of all the atoms are fixed to the values determined from the SXRD experiments.

atom	site	x	y	z	B_{iso}	occupancy
Au01	24*g*	0	0.4032(5)	0.3573(5)	0.07(4)	1
Au02	48*h*	0.1024(4)	0.3415(3)	0.1988(3)	0.07(4)	1
Au03	16*f*	0.1493(4)	0.1493(4)	0.1493(4)	0.07(4)	1
Au04	12*d*	0	0.4074(10)	0	0.94(15)	0.605
Ga04	12*d*	0	0.4074(10)	0	0.94(15)	0.395
Dy05	24*g*	0	0.1879(3)	0.3063(2)	0.20	1
Ga06	24*g*	0	0.2355(10)	0.0935(10)	0.94(15)	0.658
Au06	24*g*	0	0.2566(10)	0.0620(10)	0.94(15)	0.342
Ga07	12*e*	0.2003(8)	0	0.5	0.13(10)	1
Ga08	8*c*	0.25	0.25	0.25	0.13(10)	1
Au09	24*g*	0	0.0433	0.0912	0.07(4)	0.152
Ga10	24*g*	0	0.0912	0.0818	0.13(10)	0.182

In principle, a noncoplanar ferromagnetic (FM) structure in a cubic system cannot be uniquely determined from powder neutron diffraction (PND) alone, because FM-induced magnetic reflections strongly overlap and the measured intensities are powder-averaged. Therefore, several assumptions are required. First, the magnetic space group is assumed to be $R\bar{3}$, consistent with previous reports[7-10], which imposes an intrinsic threefold symmetry. Notably, $R\bar{3}$ contains two inequivalent magnetic sites. Second, the magnitudes of the magnetic moments are assumed to be equal for all Dy sites. This constraint can be satisfied by using the basis vectors of IR8 under appropriate restraints. With these constraints, three adjustable parameters remain in the refinement, leading to two candidate magnetic structures. As summarized in Table S15, both models provide reasonable fits to the data; however, one model yields a small component perpendicular to the local mirror plane, whereas the other produces a much larger component. The former is considered more plausible, as the resulting easy-axis direction is closer to that observed in $Au_{68}Ga_{18}Dy_{14}$. The refinement (solid black curve in Fig. 4(d) in the main text) and the corresponding magnetic structure (Fig. 4(e) in the main text) are shown. In this model, the moment magnitude is $4.61(18)\ \mu_B$, and the canting angle relative to the pseudo-fivefold axis is $67.6(2)^\circ$.

Table S15. Refined crystallographic data of two possible candidate structures.

	FM1	FM2
$C_{\mathrm{FM},1}$ (μ_{B})	2.52(29)	4.88(17)
$C_{\mathrm{FM},2}$ (μ_{B})	4.52(17)	1.60(40)
$C_{\mathrm{FM},3}$ (μ_{B})	−0.92(35)	−0.42(35)
R_{p} (%)	18.30	18.40
R_{wp} (%)	18.90	19.00
R_{e} (%)	10.84	10.88
χ^2	3.04	3.04
R_{mag}	9.53	10.9

Appendix

Crystal Electric Field Calculation Details

To quantitatively describe the crystal electric field (CEF) excitations observed in the inelastic neutron scattering spectra, calculations were performed using a point-charge model constructed from the refined atomic structure of $Au_{68}Ga_{18}Dy_{14}$. In this model, the electrostatic potential acting on the Dy^{3+} ion is generated by the surrounding atomic charges and expanded in terms of spherical tensor operators.

The CEF potential is expressed as:

$$V_{\mathrm{CEF}} = \sum_{k=0,2,4,6} \sum_{q=-k}^{k} \langle r^k \rangle q_{kq} c_q^{(k)}(\theta, \phi) \tag{A1}$$

where $\langle r^k \rangle$ are the radial expectation values of the Dy^{3+} 4*f* wavefunction and $c_q^{(k)}$ are spherical tensor operators defined as:

$$c_q^{(k)}(\theta, \phi) = \sqrt{\frac{4\pi}{2k+1}} Y_q^{(k)*}(\theta, \phi) \tag{A2}$$

The lattice-dependent coefficients q_{kq} are given by:

$$q_{kq} \equiv \sqrt{\frac{4\pi}{2k+1}} \sum_i \frac{q_i e^2}{R_i^{k+1}} Y_q^{(k)*}(\theta, \phi) \tag{A3}$$

where *e* denotes the elementary charge, q_i is the effective charge of the *i*th atom in units of *e*, and (R_i, θ_i, ϕ_i) represents the position of the *i*th atom relative to the Dy^{3+} ion in spherical coordinates.

The radial expectation values for the Dy^{3+} 4*f* wavefunction were taken from Dirac–Fock calculations[11]:

$\langle r^2 \rangle$ = 0.2188 Å^2,
$\langle r^4 \rangle$= 0.1180 Å^4,
$\langle r^6 \rangle$ = 0.1328 Å^6.

The matrix elements of the spherical tensor operator were evaluated using the Wigner–Eckart theorem:

$$\langle J, J_{z1} | c_m^{(k)} | J, J_{z2} \rangle = (-1)^{J-J_{z1}} \times \begin{pmatrix} J & k & J \\ -J_{z1} & q & J_{z2} \end{pmatrix} \langle J \| c^{(k)} \| J \rangle \tag{A4}$$

where $\langle J \| c^{(k)} \| J \rangle$ (k = 2, 4, 6) corresponds to the reduced matrix element for Dy^{3+} ions, $-4\sqrt{17/105}$, $-16/33\ \sqrt{323/91}$, and $20/143\ \sqrt{1615/39}$.

The inelastic neutron scattering intensity associated with the CEF excitations was calculated using:

$$\left(\frac{d^2\Omega}{d\sigma dE}\right) = C \sum_{J_{z1},J_{z2}} \sum_{\mu=x,y,z} P_{J,J_{z2}} \left|\langle J, J_{z1} | J_\mu^2 | J, J_{z2} \rangle\right|^2 \times \delta(\omega - E_{J,J_{z1}} + E_{J,J_{z2}}), \quad \text{(A5)}$$

where C is a proportionality constant that includes the magnetic form factor, and $P_{J,J_{z2}}$ denotes the occupation probability of the eigenstate, $|J, J_{z2}\rangle$.

To account for chemical disorder at the Au/Ga sites surrounding the Dy ion, statistical sampling of atomic configurations was employed. For sites within R < 8 Å of the Dy^{3+} ion, Au and Ga atoms were randomly assigned according to their refined site occupancies. For more distant sites (8 Å < R < 30 Å), occupancy-weighted average charges were used to reduce computational cost. The calculated spectra therefore represent averages over 5000 randomly generated atomic configurations.

The point-charge parameters were treated as adjustable quantities and optimized to reproduce the experimental inelastic neutron spectra. The best agreement with the experimental data was obtained using

$q_{\mathrm{Au}} = 0.08$, $q_{\mathrm{Ga}} = 0.44$, $q_{\mathrm{Dy}} = -0.08$.

Using these parameters, the distribution of CEF energy levels was calculated for each randomly generated atomic configuration. The resulting statistical distribution of the Dy^{3+} CEF levels is shown in Figure S12. Because Dy^{3+} is a Kramers ion (J = 15/2), all CEF levels remain doubly degenerate. The calculated distribution demonstrates that the lowest-energy transitions correspond to excitations from the ground-state doublet to the first excited doublet, consistent with the experimentally observed peak at approximately 3.3 meV.

Finally, the principal axis of the magnetic-moment distribution was estimated by diagonalizing the expectation-value matrix:

$$\begin{pmatrix} \langle J_x^2 \rangle & \langle J_x J_y \rangle & \langle J_x J_z \rangle \\ \langle J_y J_x \rangle & \langle J_y^2 \rangle & \langle J_x J_y \rangle \\ \langle J_z J_x \rangle & \langle J_z J_y \rangle & \langle J_z^2 \rangle \end{pmatrix} \quad \text{(A6)}$$

The eigenvector associated with the largest eigenvalue defines the easy-axis direction imposed by the CEF potential.

References:

(1) Thilakan, K. K.; Denoel, F.; Huang, Y.; Fabelo, O.; Mathieu, R.; G´omez, C. P.; Sahlberg, M. Single-Crystal Neutron Diffraction Study on the $Ho_{13.6}Au_{61.1}Al_{25.3}$ Quasicrystal Approximant. *J. Alloys Compd.* **2024**, *1002*, 175263.
(2) Tanaka, K.; Tanaka, Y.; Ishimasa, T. Tsai-Type Quasicrystal and Its Approximant in Au Al Tm Alloys. *Acta Phys. Pol. A* **2014**, *126* (2), 603–607.
(3) Nawa, K.; Okuyama, D.; Ebina, A.; Sato, T. J.; Ishikawa, A.; Tamura, R. Single-Crystal Neutron Diffraction Study on the Quasicrystal Approximant $Au_{70}Al_{16}Tb_{14}$. *J. Phys. Conf. Ser.* **2023**, *2461*, 012015.
(4) Sato, T. J.; Ishikawa, A.; Sakurai, A.; Hattori, M.; Avdeev, M.; Tamura, R. Whirling Spin Order in the Quasicrystal Approximant $Au_{72}Al_{14}Tb_{14}$. *Phys. Rev. B* **2019**, *100*, 054417.
(5) Kikugawa, N.; Hiroto, T.; Ishikawa, A.; Suzuki, S.; Sakurai, H.; Tamura, R. Magnetocaloric Effect in Ferromagnetic 1/1 Quasicrystal Approximants $Au_{64}Al_{22}R_{14}$ (R = Gd, Tb, and Dy). *J. Alloys Compd.* **2021**, *882*, 160669.
(6) Suzuki, S.; Ishikawa, A.; Yamada, T.; Sugimoto, T.; Sakurai, A.; Tamura, R. Magnetism of Tsai-Type Quasicrystal Approximants. *Mater.*

Trans. **2021**, *62* (3), 298–306.

(7) Hiroto, T.; Sato, T. J.; Cao, H.; Hawai, T.; Yokoo, T.; Itoh, S.; Tamura, R. Noncoplanar Ferrimagnetism and Local Crystalline-Electric-Field Anisotropy in the Quasicrystal Approximant $Au_{70}Si_{17}Tb_{13}$. *J. Phys. Condens. Matter* **2020**, *32*, 415802.

(8) Hiroto, T.; Tokiwa, K.; Tamura, R. Sign of Canted Ferromagnetism in the Quasicrystal Approximants Au-SM-R (SM = Si, Ge and Sn / R = Tb, Dy and Ho). *J. Phys. Condens. Matter* **2014**, *26* (21), 216004.

(9) Gebresenbut, G. H.; Shiino, T.; Andersson, M. S.; Qureshi, N.; Fabelo, O.; Beran, P.; Qvarngård, D.; Henelius, P.; Rydh, A.; Mathieu, R.; Nordblad, P.; Pay Gomez, C. Effect of Pseudo-Tsai Cluster Incorporation on the Magnetic Structures of R-Au-Si (R = Tb, Ho) Quasicrystal Approximants. *Phys. Rev. B* **2022**, *106*, 184413.

(10) Labib, F.; Nawa, K.; Suzuki, S.; Wu, H.; Ishikawa, A.; Inagaki, K.; Fujii, T.; Sato, T. J.; Tamura, R. Unveiling Exotic Magnetic Phase Diagram of a Non-Heisenberg Quasicrystal Approximant. *Mater. Today Phys.* **2024**, *40*, 101321.

(11) Freeman, A. J.; Desclaux, J. P. Dirac-Fock Studies of Some Electronic Properties of Rare-Earth Ions. *J. Magn. Magn. Mater.* **1979**, *12* (1), 11–21.
